# Overcoming endocrine resistance due to reduced PTEN levels in estrogen receptor-positive breast cancer by co-targeting mammalian target of rapamycin, protein kinase B, or mitogen-activated protein kinase kinase

**DOI:** 10.1186/s13058-014-0430-x

**Published:** 2014-09-11

**Authors:** Xiaoyong Fu, Chad J Creighton, Nrusingh C Biswal, Vijetha Kumar, Martin Shea, Sabrina Herrera, Alejandro Contreras, Carolina Gutierrez, Tao Wang, Sarmistha Nanda, Mario Giuliano, Gladys Morrison, Agostina Nardone, Kristen L Karlin, Thomas F Westbrook, Laura M Heiser, Pavana Anur, Paul Spellman, Sylvie M Guichard, Paul D Smith, Barry R Davies, Teresa Klinowska, Adrian V Lee, Gordon B Mills, Mothaffar F Rimawi, Susan G Hilsenbeck, Joe W Gray, Amit Joshi, C Kent Osborne, Rachel Schiff

**Affiliations:** 1 Lester and Sue Smith Breast Center, Baylor College of Medicine, 1 Baylor Plaza, Houston, 77030 TX USA; 2 Dan L. Duncan Cancer Center, Baylor College of Medicine, 1 Baylor Plaza, Houston, 77030 TX USA; 3 Department of Medicine, Baylor College of Medicine, 1 Baylor Plaza, Houston, 77030 TX USA; 4 Department of Molecular and Cellular Biology, Baylor College of Medicine, 1 Baylor Plaza, Houston, 77030 TX USA; 5 Department of Radiology, Baylor College of Medicine, 1 Baylor Plaza, Houston, 77030 TX USA; 6 Department of Pathology, Baylor College of Medicine, 1 Baylor Plaza, Houston, 77030 TX USA; 7 Department of Biochemistry and Molecular Biology, Baylor College of Medicine, 1 Baylor Plaza, Houston, 77030 TX USA; 8 Department of Molecular and Human Genetics, Baylor College of Medicine, 1 Baylor Plaza, Houston, 77030 TX USA; 9 Department of Biomedical Engineering, Oregon Health and Science University, 3181 SW Sam Jackson Park Road, Portland, 97239 OR USA; 10 Department of Molecular and Medical Genetics, Oregon Health and Science University, 3181 SW Sam Jackson Park Road, Portland, 97239 OR USA; 11 AstraZeneca Oncology iMed, 35 Gatehouse Dr, Waltham, 02451 MA USA; 12 AstraZeneca Oncology iMed, Alderley Park, Macclesfield, Cheshire SK10 4TG UK; 13 Department of Pharmacology and Chemical Biology, Women’s Cancer Research Center, University of Pittsburgh Cancer Institute, 5150 Centre Avenue, Pittsburgh, 15232 PA USA; 14 Department of Systems Biology, M.D. Anderson Cancer Center, 1515 Holcombe Boulevard, Houston, 77030 TX USA; 15 Department of Clinical Medicine and Surgery, University of Naples Federico II, Corso Umberto I, 40, Naples, 80138 Italy

## Abstract

**Introduction:**

Activation of the phosphatidylinositol 3-kinase (PI3K) pathway in estrogen receptor α (ER)-positive breast cancer is associated with reduced ER expression and activity, luminal B subtype, and poor outcome. Phosphatase and tensin homolog (PTEN), a negative regulator of this pathway, is typically lost in ER-negative breast cancer. We set out to clarify the role of reduced PTEN levels in endocrine resistance, and to explore the combination of newly developed PI3K downstream kinase inhibitors to overcome this resistance.

**Methods:**

Altered cellular signaling, gene expression, and endocrine sensitivity were determined in inducible PTEN-knockdown ER-positive/human epidermal growth factor receptor 2 (HER2)-negative breast cancer cell and/or xenograft models. Single or two-agent combinations of kinase inhibitors were examined to improve endocrine therapy.

**Results:**

Moderate PTEN reduction was sufficient to enhance PI3K signaling, generate a gene signature associated with the luminal B subtype of breast cancer, and cause endocrine resistance *in vitro* and *in vivo*. The mammalian target of rapamycin (mTOR), protein kinase B (AKT), or mitogen-activated protein kinase kinase (MEK) inhibitors, alone or in combination, improved endocrine therapy, but the efficacy varied by PTEN levels, type of endocrine therapy, and the specific inhibitor(s). A single-agent AKT inhibitor combined with fulvestrant conferred superior efficacy in overcoming resistance, inducing apoptosis and tumor regression.

**Conclusions:**

Moderate reduction in PTEN, without complete loss, can activate the PI3K pathway to cause endocrine resistance in ER-positive breast cancer, which can be overcome by combining endocrine therapy with inhibitors of the PI3K pathway. Our data suggests that the ER degrader fulvestrant, to block both ligand-dependent and -independent ER signaling, combined with an AKT inhibitor is an effective strategy to test in patients.

**Electronic supplementary material:**

The online version of this article (doi:10.1186/s13058-014-0430-x) contains supplementary material, which is available to authorized users.

## Introduction

The phosphatidylinositol 3-kinase (PI3K) pathway is frequently altered in breast cancer, with more than 70% of tumors displaying genetic aberrations in at least one component of this pathway [1]. Molecular profiling stratifies estrogen receptor (ER)-positive (+) breast cancer into luminal A and luminal B subtypes. The luminal B subtype is more aggressive and more resistant to endocrine therapy [2]. We have previously shown that PI3K pathway activation signatures in ER+ breast cancer are associated with reduced ER and classical ER activity, luminal B subtype, and worse outcome [3]. Furthermore, PI3K downstream signaling phosphorylates and activates ER and co-activators such as steroid receptor coactivator-3 (SRC3) in a ligand-independent manner, rendering this receptor unresponsive to tamoxifen or aromatase inhibitors [4]. Surprisingly, oncogenic mutations in *PIK3CA*, the gene encoding the catalytic subunit of PI3K, are associated with the luminal A subtype, high ER, and better clinical outcome in ER+ breast cancer patients receiving endocrine therapy [5],[6]. These mutations are associated with only weak activation of the PI3K pathway, probably due to subsequent negative-feedback regulation. On the other hand, loss of phosphatase and tensin homolog (PTEN), a negative regulator of the PI3K pathway, activates downstream protein kinase B (AKT)/mammalian target of rapamycin (mTOR) signaling and may contribute to endocrine therapy resistance [7]-[9]. Interestingly, we and others have reported that even moderate reduction in PTEN determined by reverse-phase protein array (RPPA) is associated with PI3K downstream pathway activation [3],[6]. Furthermore, PTEN dose-dependency was highlighted in a PTEN hypomorphic mouse model showing that subtle downregulation of PTEN by only 20% can lead to breast tumors with high penetrance [10]. Typically lost in ER-negative breast cancer, PTEN protein is decreased in more than 50% of ER+ breast cancer [6]. A recent TCGA (The Cancer Genome Atlas) study showed that *PTEN* gene aberrations (mutations or loss) are twice as frequent in luminal B as in luminal A breast cancer (24% vs*.* 13%). In contrast, as found in the previous studies mentioned above [5],[6], frequency of *PIK3CA* mutations was shown to be higher in luminal A than in luminal B breast cancer [11].

Therefore, we hypothesized that a moderate reduction in PTEN contributes to the endocrine resistance seen in the luminal B subtype via activation of the PI3K pathway. We further hypothesized that using the ER degrader fulvestrant to inhibit both ligand-dependent and -independent receptor activation combined with inhibitors of PI3K downstream signaling would be most effective in overcoming this type of resistance.

In order to fully address the impact of reduced PTEN levels on PI3K activation and endocrine response, we created models of ER+/human epidermal growth factor receptor 2 (HER2)-negative (−) breast cancer with reduced PTEN by inducible knockdown (KD). We found that moderate reductions in PTEN, commonly seen in ER+ breast cancer, activate the PI3K pathway, and reduce ER level and classical transcriptional activity. PTEN-KD generates a gene expression signature similar to luminal B breast cancer, leading to resistance to endocrine therapy *in vitro* and *in vivo*. Fulvestrant combined with an AKT inhibitor alone or combined with a mitogen-activated protein kinase kinase (MEK) inhibitor is the optimal approach to overcome the resistance.

## Methods

### Cell culture and reagents

Human breast cancer cell lines MCF7L (from Dr. Marc Lippman) and BT483 and T47D (both from the American Type Culture Collection) were all authenticated and maintained in RPMI/1640 medium supplemented with 10% heat-inactivated fetal bovine serum (FBS) and 1% penicillin/streptomycin/glutamine, and incubated at 37°C in 5% CO_2_. The mTOR inhibitor (AZD2014), AKT inhibitor (AZD5363), MEK inhibitor (selumetinib (AZD6244, ARRY-142886)), and fulvestrant were provided by AstraZeneca. β-estradiol (E2) and 4-OH-tamoxifen for *in vitro*, and tamoxifen citrate for *in vivo* experiments were purchased from Sigma-Aldrich (St Louis, MO, USA).

### pINDUCER lentiviral system

All procedures were done as described [12]. Live animal imaging was performed as previously described [13].

### Cell growth assay

Cells were pre-treated in phenol-red free (PRF) medium with 5% charcoal stripped-FBS (CS-FBS) and -/+doxycycline (Dox) three days before treatment for an additional five to ten days, as indicated. The starting number of cells in each experiment was identical across all treatment groups. Culture medium with drugs was replaced every three days. Cell growth was measured either by colorimetric methylene blue staining [14] every two days or daily real-time *in situ* cell cytometry (Celigo, Nexcelom Bioscience, Lawrence, MA, USA). Cell growth under estrogen (E2) was set as the normalization control. All the anti-estrogen treatments of estrogen deprivation (ED), tamoxifen (Tam), and fulvestrant (Ful) were in the absence of E2. The relative cell growth was determined by (cell number or OD at day_n_ - cell number or OD at day_0_) _Treatment_/(cell number or OD at day_n_ - cell number or OD at day_0_) _E2_ × 100%.

### Immunoblotting assay

This assay was performed as described previously [14]. Primary antibodies used in this study are: ER (6F11) from Abcam, Cambridge, MA, USA; PR (sc-7208) and BCL2 (sc-509) from Santa Cruz Biotechnology, Santa Cruz, CA, USA; P-AKT-Thr308 (#2214) and P-AKT-Ser473 (#2118) from Epitomics, Burlingame, CA, USA; P-PRAS40-Thr246 (#2997), P-GSK3-Ser9 (#9323), P-ERK1/2-Thr202/Tyr204 (#9101), P-S6-Ser240/244 (#2211), P-4EBP1-Thr37/46 (#9459), P-mTOR-Ser2448 (#2971), AKT (#9272), ERK1/2 (#9102), GSK-3β (#9832), PTEN (#9559), β-actin (#4970), and c-PARP (#9541) from Cell Signaling Technology, Danvers, MA, USA. All our shown Western blotting images are from the same gel with the same exposure to allow for a complete comparison between lines and across treatments.

### Quantitative reverse transcription-polymerase chain reaction (qRT-PCR)

The assay procedure was described previously [3]. Target primer sequences are as follows: *PTEN* forward GTAACGACTTCTCCATCTC, reverse ATCCACAGCAGGTATTATG; *GREB1* forward TCATCTTGTTCATCTTGTTCAGT, reverse GCATCTCAACCTTCTCATCTT; *BCL2* forward GGGGAGGATTGTGGCCTTC, reverse CAGGGCGATGTTGTCCACC; *ER-α* forward AACCGAGATGATGTAGCCAGC, reverse CAGGAACCAGGGAAAATGTG; *PR* forward GATGCTGTATTTTGCACCTGATCTA, reverse GAACTCTTCTTGGCTAACTTGAAGCT; *CAV1* forward GGTCAACCGCGACCCTAAA, reverse CCTTCCAAATGCCGTCAAA; *GAPDH* forward AAGGTGAAGGTCGGAGTC, reverse GGGGTCATTGATGGCAAC.

### Cell cycle and apoptosis flow cytometry

Cells were trypsinized and stained with SytoxBlue (Invitrogen, Carlsbad, CA, USA) to distinguish the dead cells. Vybrant DyeCycle Ruby stain (Invitrogen) was applied to quantify DNA in living cells for 30 minutes in 37°C. Stained cells were analyzed by flow cytometry (LSRFortessa, BD Biosciences, Franklin Lakes, NJ, USA) using filter1 (Em: 680 nm) for signal acquisition, and analysis was done by using Flowjo (v9) software (Tree Star Inc, Ashland, OR, USA). After 48 hours of treatment, cells were trypsinized and stained with Annexin V-APC (Invitrogen) and SytoxBlue (Invitrogen) for 15 minutes before being analyzed by flow cytometry (LSRFortessa, BD Biosciences) using filter1 (Em: 680 nm) and filter2 (Em: 480 nm) for Annexin V and SytoxBlue staining, respectively. Data analysis was done by using Flowjo (v9) software (Tree Star).

### Colony and tumorsphere formation assay

MCF7L-shPTEN cells were seeded at 6,000 per 6-cm dish and treated as before. After three weeks of endocrine treatment, colonies were stained by crystal violet and counted by ImageJ software [15]. BT483-shPTEN cells were seeded at 3,000 per well in a low-adhesive 96-well plate and prepared as before. The tumorspheres were scanned and counted by *in situ* cell cytometry two weeks after endocrine treatment (diameter >50 μm as threshold), according to the manufacturer’s instruction.

### Immunohistochemical (IHC) staining

This assay was performed as described previously [14]. Briefly, paraffin-embedded blocks of xenograft tumor tissues were organized into a 3-mm core tissue array and IHC staining was performed on 3-micron sections from these arrays. By using a PTEN index array as an internal validation control, an optimized protocol for PTEN IHC staining was developed and followed. Briefly, freshly cut slides were deparaffinized and subjected to epitope retrieval in 0.1 M Tris-HCl buffer (pH 9.0). After blocking in 3% hydrogen peroxide for 5 minutes, slides were incubated with PTEN antibody (#9188, Cell Signaling Technology) at a dilution of 1:100 for one hour. Immunodetection was performed with the EnVision + System (Dako Cytomation, Carpinteria, CA, USA). The cytoplasmic staining of PTEN or pAKT in tumors was scored by Allred score [16]. Ki67 was scored by percentage of positive cells.

### Gene expression analysis

The original RNA-seq data of MCF7L-shPTEN cells were deposited into the GEO database (GSE53300). TCGA data were accessed from the TCGA data portal [11],[17]. The mega-set Compendium of breast cancer gene expression profiles was previously reported [18]. RNA samples were extracted by using Qiagen (Germantown, MD, USA) RNeasy Mini kit and labeled with Illumina (San Diego, CA, USA) TruSeq RNA kit. Next-generation RNA-seq was performed in Illumina RNA-seq platform and scanned by HiSeq 2000. We used the freely available Cufflinks/Cuffdiff software package (v1.3.0) to identify differences in expression of genes/isoforms between the two samples [19]. In brief, this approach employs a statistical approach based on Jensen-Shannon divergence, looking for differences in the distribution of expression of isoforms between the sample sets. Differentially expressed genes between PTEN-wild-type (WT) and -KD cells were chosen by FDR <0.05 and their values were represented by Java TreeView [20]. Pearson’s correlation (represented as a *t* statistic or `*t* score’) was used as previously described [21],[22], in order to assess the global similarity of gene patterns between PTEN-low and other known gene signatures. Gene set enrichment analysis was performed by one-sided Fisher’s exact test (represented as two-sided Fisher’s *z* score). To score each human breast tumor expression profile for similarity to the PTEN-low gene signature, a `*t* score’ was derived for the tumor in relation to the PTEN-low signature patterns, as previously described [21],[22].

### Drug interaction test

Enhancement (or attenuation) of growth inhibition with combination treatment was examined by comparing combinations to the growth inhibition of each treatment/dose as a single agent within the clinically relevant range. Briefly, two-agent combinations of kinase inhibitors were explored in 6 x 6 or 4 x 6 dose matrices, under ED or Tam with or without PTEN-KD. Each experiment was run in quadruplicate. Cell growth under ED (+/-PTEN-KD) or Tam (+/-PTEN-KD) alone was set as the reference (inhibition rate of 0) and used to compute growth inhibition for single-agent and combination doses of pairs of kinase inhibitors. For display purposes as a heatmap, average dose-specific growth inhibition in each dose/drug matrix was scaled so as to not exceed 100% by dividing by the maximum growth inhibition. In addition, in each dose matrix we used the Min test of Laska and Meisner [23], implemented as two one-tailed *t* tests to compare each combination to the growth inhibition at corresponding single-agent doses. The *P* value for the Min test is the maximum of the two separate one-tailed *P* values. A significant Min test *P* value (*P* ≤0.05) indicates that the combination is superior to both of the single agents at the same doses, and suggests enhanced efficacy. Since *P* values are one-tailed, Min test *P* values that are equal to or exceed 0.95 indicate that the combination is worse (that is has less effect) than at least one of the single agents, and suggest an attenuation of effect.

### Animal studies

Animal care and animal experiments were in accordance with and approved by the Baylor College of Medicine Institutional Animal Care and Use Committee (IACUC). MCF7L-shPTEN xenografts were established in ovariectomized five- to six-week-old athymic mice (Harlan Laboratories, Indianapolis, IN, USA) supplemented with E2 pellets by inoculating 6 × 10^6^ cells subcutaneously as described previously [24]. When tumors reached the volume of 200 mm^3^ (two to three weeks), mice bearing the MCF7L-shPTEN xenografts were randomized to continuing E2 pellets, or E2 pellets withdrawn (ED) alone or in combination with Tam or Ful, as previously described [25]. All arms were fed -/+Dox (200 μg/ml) in the drinking water [26]. In a separate experiment, mice bearing MCF7L-shPTEN xenografts were randomized to E2, E2 plus an AKT inhibitor (i) (120 mg/kg, twice daily by gavage), Ful, or Ful plus AKTi, all arms +Dox. Each arm contained a minimum of 10 mice. Tumor diameters were measured once or twice weekly and tumor volume (vol) was calculated as vol = (width)^2^ × length/2 [27]. Mice were sacrificed two weeks after treatment or when tumors reached the volume of 1,000 mm^3^. Tumor tissues were removed and embedded in paraffin or snap-frozen in liquid nitrogen for later use.

### Statistical analysis

All the statistical analysis was based on the quadruplicated data. All *in vitro* experiments were repeated at least three times. Quantitative data are shown as mean ± standard errors (SE). Significant difference (*P* <0.05) was determined by ANOVA or Bonferroni *post hoc* tests (multiple testing corrected). We used R software (v2.13.0) for box plot and GraphPad Prism (v5.04) (GraphPad Software Inc, La Jolla, CA, USA) for statistical analysis. The Kaplan-Meier survival and Wilcoxon tests were used for *in vivo* xenograft tumor analysis.

## Results

### Inducible PTEN-KD enhances PI3K signaling, decreases ER levels and activity, and generates a gene expression profile associated with the luminal B subtype

**Figure 1:**
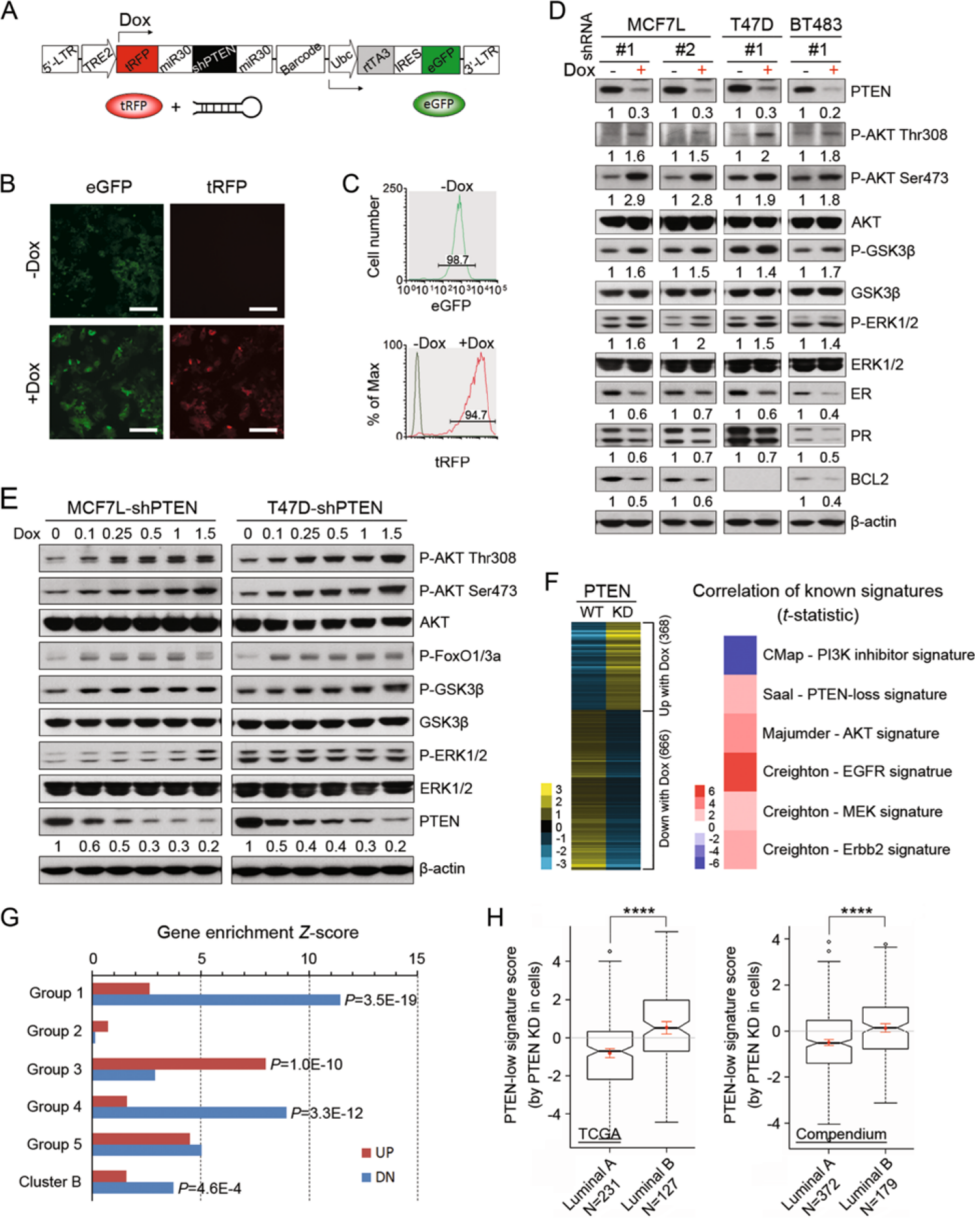
**PTEN-KD enhances PI3K signaling, reduces expression of ER and its regulated genes, and promotes expression profile of genes associated with luminal B subtype. (A)** Diagram of the lentiviral pINDUCER vector. **(B)** Fluorescence microscopy (20×) shows the eGFP and tRFP expression in MCF7L-shPTEN cells after Dox induction for 48 h. **(C)** The quantification of the positive population of eGFP and tRFP cells was analyzed by flow cytometry. **(D)** After three days of Dox induction, the cell lysates of shPTEN cell models were subjected to Western blotting. Numbers under each blot indicate protein densitometry normalized to β-actin (values in -Dox cells were set as 1). **(E)** Cell lysates from MCF7L-shPTEN and T47D-shPTEN cells after five days induction with a range of Dox doses were blotted by the indicated antibodies (densitometry shown under the PTEN blotting). **(F)** PTEN-low gene signature derived from PTEN-KD cells representing the differentially expressed genes compared to -WT cells was correlated with known gene signatures related to growth factor signaling [28]-[30]. Heat map of *t* statistic indicates the global similarity between signatures based on the Pearson’s correlations. **(G)** Up- or downregulated (DN) genes in PTEN-KD cells were analyzed for gene enrichment (one-sided Fisher’s exact test) in the gene sets of endocrine resistant xenograft tumors (Group 1 to 5) [31] or E2-induced MCF7 cells (Cluster B) [30]. **(H)** Box plot shows the PTEN-low gene signature scores of each ER+ luminal tumor from datasets of TCGA [11] and Compendium [18]. The mean value ± standard deviation of all samples in each subtype is marked within the box plot in red (^****^P <0.0001, Student’s *t* test). Dox, doxycycline; E2, β-estradiol; ED, estrogen deprivation; eGFP, enhanced GFP; ER, estrogen receptor α; KD, knockdown; PI3K, phosphatidylinositol 3-kinase; PTEN, phosphatase and tensin homolog; TCGA, The Cancer Genome Atlas; tRFP, turbo-RFP.

To model reduced PTEN in ER+ breast cancer *in vitro*, we established stable clones of several ER+ breast cancer cell lines transduced with a Dox-inducible PTEN short-hairpin RNA (shRNA) lentiviral system (pINDUCER) [12]. As depicted in Figure 1A, the pINDUCER integrates both enhanced-GFP (eGFP) and turbo-RFP (tRFP) to facilitate flow sorting and real-time monitoring of shRNA expression, respectively. After testing four different PTEN-shRNA sequences from pGIPZ clones (Open Biosystems, Huntsville, Al, USA) (Additional file 1: Figure S1A and B), we selected two validated shRNA sequences (#1: V2LHS_231477 and #2: V2LHS_92317) to construct the pINDUCER-shPTEN lentiviral vector. The stable MCF7L-shPTEN cell model was established by lentiviral infection at MOI (multiplicity of infection) of 0.8, followed by eGFP sorting selection (Additional file 1: Figure S1C). As expected, the sorted cells with high-eGFP intensity showed enhanced KD efficiency as measured by PTEN Western blotting (Additional file 1: Figure S1D). To model reduced but not absent PTEN, we used the cell population with low-eGFP intensity for subsequent experiments. After induction for 48 hours, the PTEN-KD cells expressing tRFP account for 94.7% of the total cells measured by flow cytometry. In contrast, there is no detectable tRFP in the -Dox cells, which showed 98.7% eGFP positivity (Figure 1B and C).

Two additional ER+/HER2- cell models (T47D and BT483) were established by using one (#1) of the two validated shRNA sequences. Together with MCF7L-shPTEN, all three models showed the enhanced PI3K/AKT signaling upon PTEN KD indicated by the increased phosphorylation of AKT (at both Thr 308 and Ser 473) and its substrate GSK3β (Figure 1D). A modest increase in ERK1/2 phosphorylation in the same models suggested an impact of reduced PTEN on the mitogen-activated protein kinase (MAPK) pathway. On the other hand, ER and its classical downstream gene products PR and BCL2 were decreased in PTEN-KD cells. The MCF7L-shPTEN cells with two different PTEN-shRNA had similar changes in these measured proteins and the KD efficiency at mRNA level (Additional file 2: Figure S2A). None of these molecular changes were observed in the MCF7L-luciferase shRNA (shLuc) cells as the negative control (Additional file 2: Figure S2B). To better understand the relationship between PTEN level and PI3K signaling, we titered the Dox concentration to knockdown PTEN in a dose-dependent manner. Interestingly, increased phosphorylation of AKT and its substrates (FoxO1/3a and GSK3β) was observed even when PTEN was only modestly reduced by 50% in both MCF7L- and T47D-shPTEN cell models (Figure 1E). For consistency, unless otherwise specified, we used 1 μg/ml of Dox in the following experiments.

To further explore the impact of PTEN-KD on global gene expression, total RNA extracted from MCF7L-shPTEN cells (-/+Dox) was subjected to next-generation RNA sequencing (RNA-seq). Overall, there were a total of 1,034 genes (368 up, 666 down) differentially expressed in PTEN-KD cells compared to PTEN-WT cells. As expected, this PTEN-low gene signature derived from PTEN-KD is inversely correlated with the signature derived from cells treated with PI3K inhibitor (CMap dataset) [28]. Importantly, we also found that this PTEN-low gene signature is significantly correlated with several known oncogenic gene signatures from human breast cancer with complete PTEN loss [32], AKT transgenic mouse [29], or epidermal growth factor receptor (EGFR)- or ERBB2-transfected MCF7 cells (Figure 1F, *P* <0.05, Pearson’s correlations), and is closely correlated with gene signature from constitutively active MEK-transfected MCF7 cells (*P* = 0.07) [30]. Further gene enrichment analysis showed that the genes downregulated by PTEN-KD were significantly enriched for estrogen (E2)-induced genes (Cluster B) *in vitro*[30] (Figure 1G). Indeed, we confirmed that the mRNA levels of *ER*, *PR*, *BCL2*, and two additional ER-regulated genes (*GREB1* and *CAV1*) were significantly decreased in PTEN-KD cells (Additional file 3: Figure S3A). These data corroborate our previous finding that activation of PI3K signaling, here through reducing PTEN, is inversely correlated with ER levels and activity. When interrogating the gene sets associated with endocrine therapy resistance in our previously published MCF7 xenograft mouse models (Group 1 to 5) [31], we found that the genes up- or downregulated in the PTEN-KD cells were significantly enriched for the genes in Group 3 or Group 1/4, which represent the gene sets that are increased or decreased upon acquired resistance *in vivo*, respectively (Figure 1G). These findings suggest that reduced PTEN levels could contribute to resistance to endocrine therapy by reducing the level of ER and its signaling activity in addition to activation of the PI3K pathway, which mediates its own intrinsic growth- and survival-promoting signals.

Next we asked to what extent the shPTEN cell model recapitulates the influence of reduced PTEN in luminal ER+ breast cancer. We used our PTEN-low gene signature to interrogate the microarray datasets from TCGA [11] and the mega-set Compendium [18]. As shown in Figure 1H, the luminal B subtype of breast cancer has a significantly higher score than luminal A in both datasets. This difference in PTEN-low gene signature score between two luminal subtypes can be reflected at least partially by the significantly lower PTEN mRNA levels in luminal B than in luminal A subtype in both datasets (Additional file 3: Figure S3B). Since all of the cell models we used have endogenous *PIK3CA* mutations (E545K in MCF7L, H1047R in T47D, and E542K in BT483), we asked whether ER+ tumors with different PTEN levels have a distinct distribution of *PIK3CA* mutations. Within 349 ER+ tumors with matched information for both events (data from TCGA), we found that there is no significant association between *PIK3CA* mutations and PTEN mRNA levels (Additional file 4: Figure S4).

### Moderate PTEN reduction decreases sensitivity to endocrine therapies in ER+/HER2- breast cancer cells

**Figure 2:**
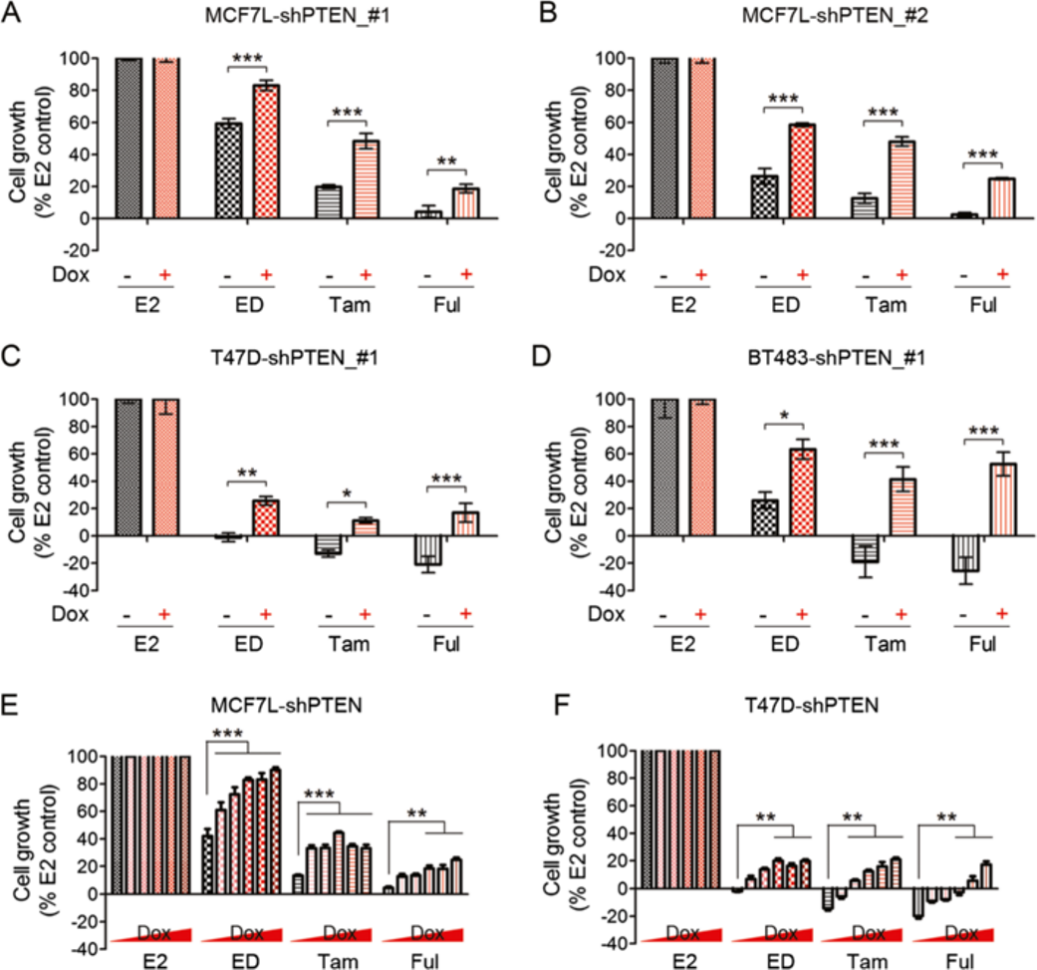
**Moderate reduction in PTEN decreases endocrine sensitivity in ER+/HER2- breast cancer cell models.** MCF7L-shPTEN cells with two different shRNA sequences (#1 and #2) **(A** **and B)**, T47D-shPTEN (#1) cells **(C)**, and BT483-shPTEN (#1) cells **(D)** were grown in PRF medium with 5% CS-FBS under -/+Dox for three days, then treated with E2 (1 nM) as control, continuing the same medium (ED), Tam (100 nM), or Ful (100 nM) for five days in 96-well plates. Cell growth (%) at day 5 in all anti-estrogen groups (ED, Tam, and Ful) was normalized to E2 -/+Dox control. There is no noticeable change in cell growth between E2-Dox and E2 + Dox groups. Cell growth was monitored daily by *in situ* cell cytometry (Celigo). **(E** **and F)** MCF7L-shPTEN and T47D-shPTEN cells were prepared as before with an additional range of Dox induction and subjected to endocrine treatment. Cell growth (%) at day 5 in all anti-estrogen groups (ED, Tam, and Ful) was normalized to E2 -/+Dox control. The Bonferroni *post hoc* test was used for all pairwise comparisons (^*^P <0.05, ^**^P <0.01, ^***^P <0.001). CS-FBS, charcoal stripped-fetal bovine serum; Dox, doxycycline; E2, β-estradiol; ED, estrogen deprivation; ER, estrogen receptor α; Ful, fulvestrant; HER2, human epidermal growth factor receptor 2; PRF, phenol-red free; PTEN, phosphatase and tensin homolog; shRNA, short hairpin RNA; Tam, tamoxifen.

To assess the effect of reduced PTEN on endocrine sensitivity, cells were treated with E2 control, ED, tamoxifen (Tam), or fulvestrant (Ful), and cell number was monitored using an *in situ* cell cytometer (Celigo, Nexcelom Bioscience). As shown in Figure 2A-D, while cell growth was strongly inhibited by anti-estrogen treatment in PTEN-WT cells, growth inhibition was substantially less in PTEN-KD cells. PTEN-KD significantly attenuated the anti-estrogen effect in blocking cell cycle S-phase entry, which partially explains the cell growth advantage over PTEN-WT cells (Additional file 5: Figure S5A). The decreased endocrine sensitivity induced by PTEN-KD was not observed in MCF7L-shLuc cells (Additional file 2: Figure S2C). Reduced endocrine sensitivity was further confirmed by colony formation assay in MCF7L-shPTEN cells, and by tumorsphere formation assay in the BT483-shPTEN model (Additional file 5: Figure S5B and C). Importantly, in both MCF7L- and T47D-shPTEN cells with dose-dependent PTEN decreasing levels as shown in Figure 1E, even modest reduction in PTEN caused attenuated growth inhibition by endocrine regimens (Figure 2E and F). Specifically, PTEN-KD by the lowest dose of Dox, in some instances, showed no difference in reducing endocrine sensitivity compared to the highest extent of PTEN-KD (Figure 2E, Tam treatment group).

### PTEN-KD leads to endocrine resistance in xenograft tumors

**Figure 3:**
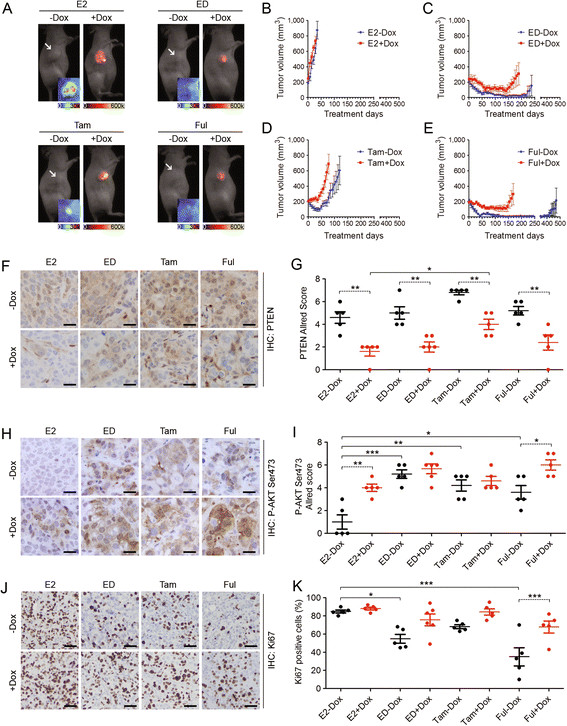
**PTEN-KD confers endocrine resistance in the xenograft mouse model. (A)** Fluorescent live images showed MCF7L-shPTEN xenograft tumors at week 6 after randomization. Fluorescent red suggests the expression of tRFP with the color bar indicated at bottom. The tumors without a red signal (arrow indicated) show the fluorescent green (eGFP expression) in the insets. **(B-E)** Growth curves of xenograft tumors under E2, ED, Tam, or Ful, all -/+Dox (n ≥10 in each arm). The IHC staining of PTEN **(F)**, pAKT-S473 **(H)**, and Ki67 **(J)** in tissue microarrays of xenograft tumors after two weeks of treatment was quantified as the Allred scores of cytoplasmic PTEN **(G)** and cytoplasmic pAKT-S473 **(I)**, or as the proportion of Ki67 positive cells **(K)**. n = 6 for each arm; scale bar, 50 μm (F and H), 100 μm (J). The Bonferroni *post hoc* test was applied for paired comparisons between -/+Dox in each endocrine group, or between the anti-estrogen and E2 groups (^*^P <0.05, ^**^P <0.01, ^***^P <0.001). Dox, doxycycline; E2, β-estradiol; ED, estrogen deprivation; eGFP, enhanced GFP; Ful, fulvestrant; IHC, immunohistochemical; KD, knockdown; PTEN, phosphatase and tensin homolog; Tam, tamoxifen; tRFP, turbo-RFP.

To determine the effect of PTEN-KD on endocrine sensitivity in an *in vivo* xenograft model, ovariectomized nude mice bearing MCF7L-shPTEN xenografts were randomized to E2, E2 withdrawal (ED), or ED combined with Tam or Ful, all with -/+Dox to modulate PTEN levels. The dual-fluorescence in the pINDUCER system allows the real-time assessment of shRNA expression as well as tumor growth by live animal imaging [12]. As shown in Figure 3A, at week 6 after randomization, there is no detectable shPTEN expression in all -Dox xenograft tumors shown by the negative tRFP signal. The positive eGFP signal confirms the persistent genome integration of the pINDUCER cassette. In contrast, all +Dox tumors expressed tRFP, which quantitatively matched the tumor size measurements. The positive tRFP signal was maintained in all palpable +Dox tumors throughout the life span of the mice, assuring the persistence of shPTEN expression in this xenograft mouse model.

As expected, all the xenografts in the E2 arm (-/+Dox) reached the 1,000 mm^3^ harvest size in less than 50 days (Figure 3B). In -Dox groups, all of the anti-estrogen arms led to significant tumor regression, and the median time to tumor regression (TTR), defined as a 50% reduction in tumor size since randomization, was 25, 24, and 17 days, respectively (Figure 3C-E; Table 1). In contrast, PTEN-KD significantly delayed tumor regression in the ED and Ful arms (Table 1, median TTR of 108 and 107 days, respectively), and caused tumors to grow without regression in the Tam arm. Also in the Tam arm, the median time to tumor progression (TTP), defined as the tumor size doubling since randomization, was significantly shortened in PTEN-KD compared to -WT tumors (Figure 3D; Table 1, 54 days vs*.* 86 days). Although at 470 days of follow-up the median TTP had not yet been achieved in ED and Ful arms, the increased progression rate was obvious in PTEN-KD versus -WT tumors (Figure 3C and E; Table 1, 43% vs*.* 13%, and 47% vs*.* 14%, respectively).

**Table 1:** **Tumor regression and progression under endocrine therapy in the MCF7L-shPTEN xenograft model with PTEN-WT (-Dox) or -KD (+Dox)**

Treatment	TR^a^(%)	TTR^b^(95% CI)	***P*** ^e^	TP470^c^(%)	TTP^d^(95% CI)	***P***
E2-Dox	0	NA^f^ (NA-NA)	1	100	8 (5–12)	0.671
E2 + Dox	0	NA (NA-NA)		100	10 (6–13)	
ED-Dox	100	25 (6–39)	0.011	13	NA (223-NA)	0.098
ED + Dox	64	108 (30-NA)		43	NA (41-NA)	
Tam-Dox	71	24 (9-NA)	0.002	71	86 (73-NA)	0.022
Tam + Dox	24	NA (43-NA)		94	54 (36–71)	
Ful-Dox	100	17 (6–24)	<0.001	14	NA (462-NA)	0.107
Ful + Dox	73	107 (38–198)		47	NA (101-NA)	

^a^TR is the fraction of mice achieving tumor regression, defined by the tumor size halving since the randomization; ^b^TTR is the median time to tumor regression with 95% confidence interval; ^c^TP470 is the fraction of mice experiencing tumor progression within 470 days after treatment, defined by the tumor size doubling since the randomization; ^d^TTP is the median time to tumor progression with 95% confidence interval; ^e^*P* value is based on the generalized Wilcoxon test comparing the -/+Dox groups in each endocrine treatment arm; ^f^NA = not-achieved. PTEN, phosphatase and tensin homolog; WT, wild-type; Dox, doxycycline; KD, knockdown; TTR, time to tumor regression; TTP, time to tumor progression; E2, β-estradiol; ED, estrogen deprivation; Tam, tamoxifen; Ful, fulvestrant.

To better understand endocrine resistance caused by reduced PTEN *in vivo*, IHC assay was performed in a tissue microarray including tumor samples collected after two weeks of treatment. An optimized PTEN IHC protocol was developed in a PTEN index-slide test (Additional file 6: Figure S6). As expected, PTEN was significantly decreased in tumors from all +Dox groups (Figure 3F and G). Of note, the positive staining was also evident in some mouse stromal cells. Interestingly, a slight increase in PTEN was observed with endocrine treatment, especially in the Tam arm of PTEN-KD tumors compared to E2 control. Phosphorylation of AKT (pAKT) at S473 was increased in PTEN-KD compared to -WT tumors, especially in the E2 and Ful arms (Figure 3H and I). An acute increase in pAKT in the first two weeks of endocrine therapy was seen in PTEN-WT tumors, but was not significant in PTEN-KD tumors, probably due to the increased level already existing in the E2 control. A modest decrease in the proliferation marker Ki67 was found in PTEN-WT tumors receiving Tam, and a significant reduction was observed in ED and Ful arms (Figure 3J and K). PTEN-KD caused an increase in Ki67 in all anti-estrogen treatment arms, and reached statistical significance in the Ful arm. These data further support the conclusion that PTEN-KD contributes to lower endocrine sensitivity and eventual progression in ER+ breast cancer.

### Kinase inhibitors downstream of PI3K enhance endocrine therapy by overcoming resistance due to reduced PTEN

**Figure 4:**
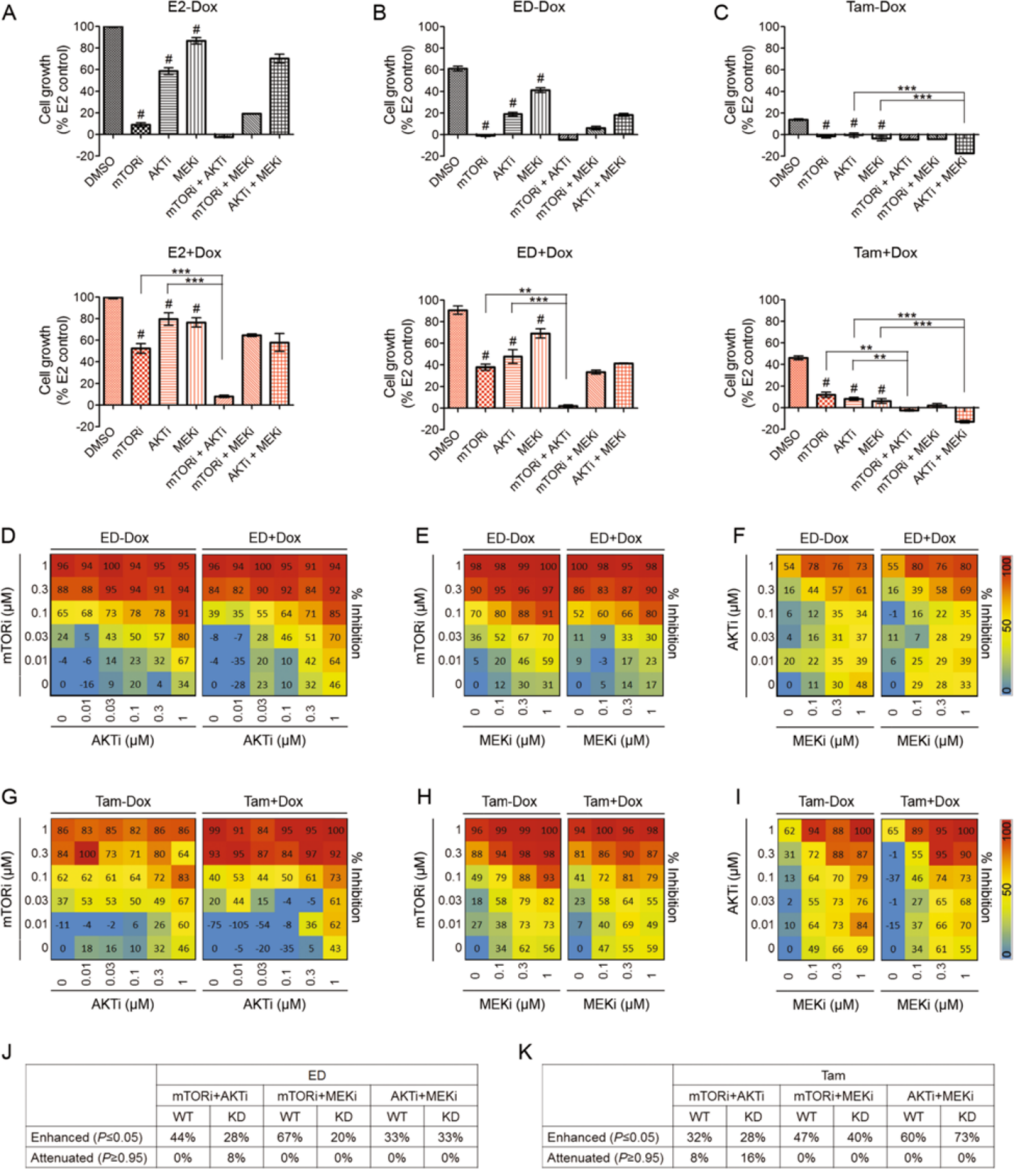
**Efficacy of combination therapy varies by PTEN levels, type of endocrine therapy, and combined kinase inhibitors.** MCF7-shPTEN cells were pre-treated in PRF medium with 5% CS-FBS and -/+Dox for three days. Bar charts presented the % of cell growth of MCF7L-shPTEN cells treated for five days with single or two-agent combination kinase inhibitors (mTORi, 0.2 μm; AKTi, 1 μm; MEKi, 1 μm), under E2 **(A)**, ED **(B)**, or Tam **(C)** condition, all -/+Dox (gray/red color). Cell growth in five days of the E2 (-/+Dox) groups was used as the normalization control. The Bonferroni *post hoc* test was performed for paired comparisons between single-kinase inhibitors and DMSO (drug carrier control) (^#^*P* <0.05), or between a two-agent kinase inhibitor combination and either agent alone (^*^P <0.05, ^**^P <0.01, ^***^P <0.001). Heat maps were used for all calculated % of growth inhibition (scaled so as to not exceed 100% by dividing by the maximum growth inhibition within each matrix) in two-agent combinations of kinase inhibitors: mTORi plus AKTi **(D** **and G)**, mTORi plus MEKi **(E and** **H)**, or AKTi plus MEKi **(F and** **I)**, all under ED or Tam. Cell growth under ED or Tam without kinase inhibitor was set as 100% (0% of growth inhibition) for the normalization control. Percentages of combinations among each matrix with enhanced (*P* ≤0.05) or attenuated (*P* ≥0.95) effect compared to single drug alone (tested by the Min test) were summarized in **(J)** (under ED) and **(K)** (under Tam). AKT, protein kinase B; CS-FBS, charcoal-stripped-FBS; Dox, doxycycline; E2, β-estradiol; ED, estrogen deprivation; Ful, fulvestrant; MEK, mitogen-activated protein kinase kinase; mTOR, mammalian target of rapamycin; PRF, phenol-red free; PTEN, phosphatase and tensin homolog; Tam, tamoxifen.

The PI3K and MAPK pathways downstream of growth factor receptors (GFRs) coordinate and crosstalk to drive cancer cell proliferation and survival. Several feedback loops have been described in which inhibition of one pathway leads to activation of the other [33],[34]. Even in the same PI3K pathway, the AKT/mTOR axis is also regulated by a feedback loop, suggesting the need for combination therapy [35]. It has been shown that pan-PI3K inhibitors exhibited preferential inhibition of tumor cells bearing *PIK3CA* mutations, while their activity in PTEN deregulated models of breast cancer is controversial [6],[36]. In this study, we focused on inhibitors of several key nodes in the PI3K and MAPK pathways including AKT, mTOR, or MEK, alone or in combination, to block compensatory pathways activated with single-agent therapy. AZD2014 and AZD5363 are ATP-competitive inhibitors that block mTORC1/2 and AKT signaling with *K*i values of 2 nM (mTOR) and <10 nM (AKT) [37],[38]. Selumetinib (AZD6244, ARRY-142886) is a non-ATP-competitive inhibitor of MEK with an IC50 for inhibition of MEK *in vitro* and in cells of approximately 12 nM [39]. The concentration chosen for the single-kinase inhibitors (i) targeting mTORC1/2 (mTORi), AKT (AKTi), or MEK (MEKi) was based on clinically relevant concentrations in patients (AstraZeneca data on file), and was validated by the decreasing phosphorylation of the kinase substrates in MCF7L cells (Additional file 7: Figure S7). Adding a single inhibitor produced significantly more growth inhibition than E2, ED, or Tam alone (Figure 4A-C). PTEN-KD attenuated the growth inhibitory effect of single inhibitors in the presence of endocrine therapy, especially for the mTORi in the ED group (by IC50 assay, data not shown). ED was less effective than Tam, alone or in combination with the inhibitors in this cell model, perhaps because signaling from the PI3K pathway can cause ligand-independent activation of the ER [40],[41].

Next we investigated combinations of two kinase inhibitors together with endocrine treatment, to block compensatory pathways. In general, combinations of the inhibitors at the specific doses were more effective in both PTEN-WT and -KD cells. The mTORi plus AKTi was highly effective in the presence of E2, ED, or Tam in PTEN-WT and -KD cells, especially the latter (Figure 4A-C). The AKTi plus MEKi was most effective with Tam and was cytotoxic even in PTEN-KD cells (Figure 4C).

To better understand drug interactions within the range of clinically relevant doses, we extended the above testing by following a 6 × 6 or 4 × 6 drug combination matrix under ED (+/-PTEN KD) or Tam (+/-PTEN KD). Figure 4D-I display the calculated average dose-specific growth inhibition in each dose/drug matrix. Using the Min test, the significance and direction of the drug interaction (that is, enhancement (*P* ≤0.05) or attenuation (*P* ≥0.95) of growth inhibition) was established for all different drug/dose combinations (Additional file 8: Figure S8) and summarized as the percentage of significant interactions within each matrix (Figure 4J-K). This analysis revealed that enhancement of inhibitory effect from any two-drug combination compared to either inhibitor alone was present within all tested drug combination matrixes (*P* ≤0.05, Min test). Within the matrix, PTEN-KD led to a decreased percentage of combinations of mTORi plus AKTi with enhanced inhibitory effect under either ED (Figure 4J) or Tam (Figure 4K), whereas the percentage of combinations with attenuated effect compared to single inhibitors (*P* ≥0.95, Min test) was increased (from 0% to 8% in ED, and from 8% to 16% in Tam). Similarly, due to PTEN-KD, the number of combinations with enhanced effect was substantially lower in mTORi plus MEKi under ED (Figure 4J, from 67% to 20%). In contrast, a slightly increased number of combinations with enhanced effect was seen in PTEN-KD cells treated with AKTi plus MEKi under Tam (Figure 4K, from 60% to 73%). These data suggest that there is drug interaction heterogeneity within the experimental settings with different types of inhibitors, PTEN status, and endocrine therapy.

### AKTi combined with fulvestrant inhibits PI3K downstream signaling, induces apoptosis, and accelerates regression of PTEN-KD xenograft tumors

**Figure 5:**
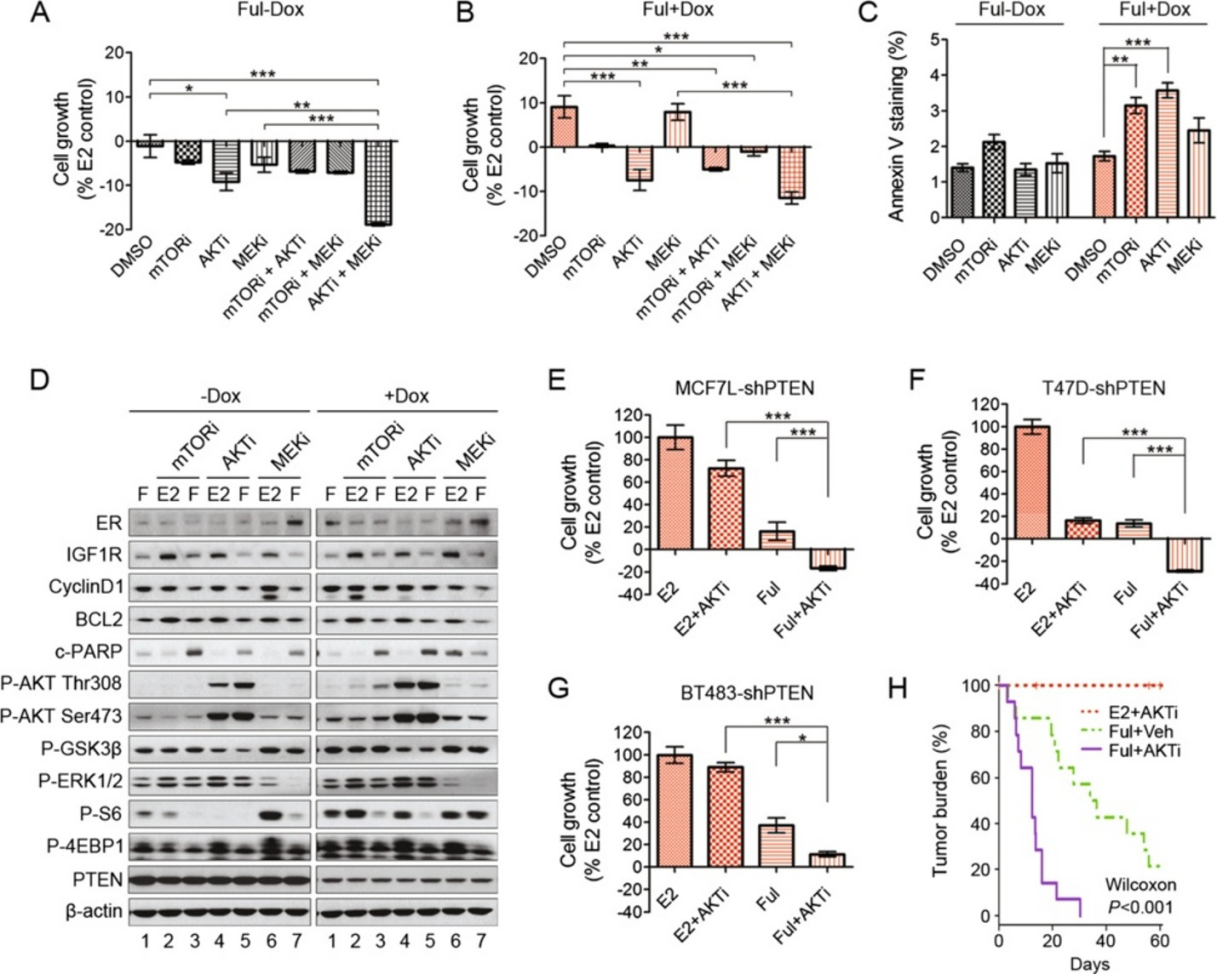
**Fulvestrant combined with the AKT inhibitor potently suppresses GFRs downstream signaling, induces apoptosis, and accelerates tumor regression. (A** **and B)** MCF7L-shPTEN cells were prepared as before and treated with Ful combined with single or two-agent kinase inhibitors, under -/+Dox (gray/red color) conditions. Cell growth was analyzed the same way as in Figure 4. **(C)** Cells were treated with Ful combined with single-kinase inhibitors (-/+Dox) for 48 hours and stained with Annexin-V-APC. Flow cytometer analysis was performed to quantify the apoptotic cells with positive staining. **(D)** MCF7L-shPTEN cells were prepared as before and then treated with Ful alone, or E2 or Ful in combination with mTORi (0.2 μm), AKTi (1 μm), or MEKi (1 μm). Cell lysates were harvested after 48 hours of treatment and immunoblotted with the indicated antibodies. Cells of MCF7L **(E)**, T47D **(F)**, and BT483 **(G)** -shPTEN models were prepared as before (all + Dox) and then treated with E2, Ful, or each in combination with AKTi (1 μm). Cell growth within five days was normalized to the E2 groups. **(H)** Kaplan-Meier plots of proportion of tumor burden without response (defined `response' as tumor size halving since randomization) within 60 days of treatment of AKTi alone in the presence of supplemented E2 pellets, or Ful combined with drug carrier (Veh) or AKTi without E2 pellets (n ≥10 in each arm). The AKTi (120 mg/kg) or drug carrier (Veh) was administered twice daily by gavage for both E2 and Ful arms. The Wilcoxon method with adjusted pairwise comparison was applied. All the pairwise comparisons of cell growth were performed by the Bonferroni *post hoc* test (^*^P <0.05, ^**^P <0.01, ^***^P <0.001). AKT, protein kinase B; Dox, doxycycline; E2, β-estradiol; Ful, fulvestrant; GFRs, growth factor receptors; MEK, mitogen-activated protein kinase kinase; mTOR, mammalian target of rapamycin; PTEN, phosphatase and tensin homolog.

Since the potent anti-estrogen Ful inhibits both ligand-dependent and -independent ER signaling, we next investigated its inhibitory effects in the absence of E2 by combining it with the various kinase inhibitors. In MCF7L cells, Ful inhibited cell growth more potently than ED or Tam in both PTEN-WT and -KD cells, although PTEN-KD decreased its activity (Figure 5A and B). Most combinations with a kinase inhibitor reduced cell number below baseline indicating a cytotoxic and not just a cytostatic effect. In the presence of Ful, at the concentrations tested the AKTi was the most potent single-kinase inhibitor in both PTEN-WT and -KD cells, and the only inhibitor that induced cytotoxic effects in the PTEN-KD cells. Of note, in PTEN-KD cells, the MEKi combined with Ful failed to induce cytotoxicity (Figure 5B). However, the addition of the MEKi to the AKTi significantly enhanced the cytotoxic effect compared to single drug alone in PTEN-WT cells, with a similar trend in the PTEN-KD cells. The combination of the AKTi or mTORi with Ful induced more apoptosis than Ful alone in PTEN-KD cells (Figure 5C).

Focusing on the MCF7L-shPTEN model, we examined the effects of the AKTi, mTORi, or MEKi, -/+Ful treatment on various signaling intermediates by Western blotting. As expected, addition of a single-kinase inhibitor effectively suppressed the phosphorylation of corresponding substrates of mTOR (S6, 4EBP1), AKT (GSK3β), and MEK (ERK1/2) after 48 hours of exposure (Figure 5D, -/+Dox, lane 3, 5, 7, compared to lane 1). Also as expected, the AKTi increased phosphorylation of AKT at T308 and S473 [37]. In PTEN-KD cells, the increased phosphorylation of AKT at T308 with the mTORi is probably due to the relief of a negative feedback loop from mTOR to inhibit IRS1-mediated PI3K signaling, which was further strengthened in cells with low PTEN (Figure 5D, lane 3 compared to lane 1) [42],[43]. Both the AKTi and the mTORi decreased phosphorylation of mTOR substrates (S6, 4EBP1), but only the AKTi kept suppressing the AKT signaling in both PTEN-WT and -KD cells, as shown by the reduced phosphorylation of GSK3β (Figure 5D, lane 3 and 5 compared to lane 1). Ful successfully suppressed ER activity, shown by the decreased levels of ER-regulated genes (IGF1R, CyclinD1, and BCL2). The combination of the mTORi or the AKTi with Ful further increased the levels of cleaved PARP (c-PARP), another apoptotic marker, compared to either agent alone in both PTEN-WT and -KD cells, especially in the latter (Figure 5D, comparison of lane 3 *vs*. lane 1 and 2; lane 5 *vs*. lane 1 and 4).

Since the AKTi, among the three inhibitors with the concentrations tested, shows the most potent efficacy as a single-kinase inhibitor when combined with Ful, we verified this combination in all three shPTEN ER + cell models. Indeed, this combination was significantly more effective than either drug alone (Figure 5E-G), though in BT483 cells, no cytotoxic effect was observed. These findings strongly suggest that an AKTi combined with Ful may be an effective therapy for ER+/HER2- breast cancer, especially when the PI3K pathway is activated by low PTEN. We further tested this hypothesis in a mouse xenograft model by focusing on the tumor response defined by tumor regression to half of the size of the tumors at randomization. We used the AKTi at 120 mg/kg, twice daily by gavage, a dose that did not cause obvious toxicity or weight loss (data not shown). Kaplan-Meier survival analysis was performed to compare tumor response in the three arms (AKTi alone, Ful alone, and the combination). A generalized Wilcoxon test showed significant differences in time to tumor halving among the three arms (Figure 5H). No tumors regressed when the AKTi was added to estrogen-treated mice. The AKTi combined with Ful, however, significantly accelerated tumor regression compared to Ful alone, with median time to tumor halving of 12 days and 35 days, respectively.

## Discussion

In this study, we provide evidence that in our model system, even partial reduction in PTEN, without complete loss, is sufficient to activate PI3K signaling, confer endocrine resistance, and trigger a gene signature that is associated with the luminal B subtype of breast cancer. In addition, we evaluated the efficacy of various combination therapies co-targeting ER and kinases downstream of PI3K signaling to treat PTEN-low ER+/HER2- breast cancer. We found that the efficacy of the different combinatorial therapies varied by the endocrine regimens and the PTEN levels. Importantly, our inducible PTEN-shRNA model system allowed us to evaluate the effects of partial PTEN reduction on endocrine resistance and to explore new strategies to overcome this resistance in the *in vivo* setting. Indeed, both our *in vitro* and *in vivo* experiments showed that AKT inhibition combined with fulvestrant is a particularly effective approach to overcome endocrine resistance due to reduced PTEN levels.

The PTEN level appears to be tightly controlled both transcriptionally and post-transcriptionally [44], with evidence showing promoter methylation in PTEN suppression in a Tam-resistant cell model [45]. Some oncogenic microRNAs have been found to target PTEN mRNA, and a recent study suggested that this regulation has prognostic potential in patients with luminal breast cancers [46]. PTEN loss (by IHC) and *PIK3CA* mutations are reported not to be mutually exclusive in breast cancers [6], suggesting that they have different contributions to tumor pathophysiology and pathway activation. We found that PTEN mRNA levels do not correlate with *PIK3CA* mutations in ER+ breast cancer, suggesting that the contribution of decreased PTEN to activation of PI3K signaling is independent of *PIK3CA* mutations. Similarly, there is no significant difference in the frequency of tumors with *PIK3CA* mutations among those tumors with the highest and lowest quartiles of PTEN protein expression measured by RPPA [11]. In fact, we found that activation of the PI3K pathway in our three ER+/HER2- cell models with mutant *PIK3CA*, was substantially enhanced upon PTEN-KD. Therefore, in light of the fact that *PIK3CA* mutations are associated with a phenotype of relatively low mTOR signaling and better outcome in ER+/HER2- breast cancer [5],[6], it may be that decreased PTEN level, and perhaps the levels of other phosphatases regulating this pathway such as INPP4B [47], represents an important mechanism resulting in the more aggressive phenotype seen in luminal B breast cancer [48].

Our data are consistent with reports that a PTEN-loss gene signature, derived from a comparison of PTEN+/− tumors by IHC, is also associated with the luminal B subtype [5] and with a poor recurrence-free survival after Tam in ER+ breast cancer patients [32]. Importantly, we showed that PTEN reduction, without complete loss, decreased the rate of tumor regression with both ED and Ful, and increased the rate of late progression. PTEN-KD led to early tumor progression with Tam treatment, suggesting that Tam may have acquired more estrogen agonist and less antagonist activity, similar to the effect we showed previously in a HER2-overexpressing xenograft model [49]. These data indicate the need for a clinically useful quantitative assay with optimized cutoffs for PTEN itself, or the establishment of other biomarkers to indicate that the PI3K pathway has been activated [50]. The failure of assays of complete PTEN loss to correlate with clinical endpoints such as therapy resistance, especially the resistance to HER2-targeted therapies [51], may be due to the fact that only modest reductions in PTEN are required to activate downstream signaling.

Our study provides a possible strategy for selecting a signal transduction inhibitor to test with an endocrine therapy in ER+ breast cancer. Numerous inhibitors of various pathways that have been implicated in endocrine resistance are in development, and it would be challenging to test all of the various combinations with different endocrine therapies in clinical trials. Here we extensively studied combinations of three inhibitors targeting the downstream signaling of the PI3K pathway with the three major types of endocrine therapy in our PTEN-KD cell models. The mTORi alone or combined with the AKTi, at the clinically relevant doses selected, was a most effective agent on a background of E2 or ED. The AKTi alone or combined with the MEKi was most effective when combined with Ful. The effectiveness of the combination of the mTORi everolimus with the aromatase inhibitor exemestane in the BOLERO-2 trial suggests that the preclinical model may have predicted correctly [52]. It will be interesting to further investigate in this study whether partial reduction in PTEN levels is related to treatment outcome. Our data also suggest that these inhibitors deserve clinical trial in patients with luminal A tumors without PI3K pathway activation, since they were effective in cells without PTEN-KD. Perhaps they were effective by blocking the PI3K pathway that can become activated by increased signaling from GFRs once ER is blocked [41].

PTEN downregulation decreases not only sensitivity to endocrine therapy, but also sensitivity to the kinase inhibitors, perhaps due to the multiple feedback loops active in this complex network that may require multiple inhibitors in combination to overcome [53],[54]. The enhanced efficacy of mTORi plus AKTi was seen under both ED and Tam, though the efficacy was attenuated at some doses by the PTEN-KD. The limited efficacy in patients treated with the mTOR inhibitor rapamycin alone may be explained by inactivating a negative feedback loop resulting in reactivation of AKT [35],[43]. A combination of mTORi plus AKTi might improve the efficacy of the mTORi alone. PTEN-KD cells were also sensitive to the combination of AKTi plus MEKi with either Tam or Ful. A synergistic effect with these inhibitors was also previously reported in a basal-like breast cancer xenograft model with either intact or deleted PTEN [55]. Therefore, optimal treatment for ER+ breast cancer with PI3K pathway activation may depend not only on the specific type of endocrine therapy, but also on the particular signaling inhibitor used to block the pathway.

Our study suggests that the most potent combination to test in patients with evidence of PI3K pathway activation may be an AKT inhibitor, together with Ful. Ful is an anti-estrogen with little or no E2-agonist activity. Furthermore, Ful is an ER degrader and would thus be more effective than ED or Tam when ER is activated in a ligand-independent manner [40]. ER and its coregulators are phosphorylated and activated by PI3K signaling, here through PTEN KD, which may convert ER from a classical ERE-mediated transcription factor to a non-classical transcription factor mediated by its binding to other sites on DNA [40],[41]. This suggests that in ER+ breast cancer with high PI3K signaling, downregulation of ER may be the preferred endocrine therapy. Other studies using the long-term estrogen-deprivation (LTED)-resistant models with increased PI3K signaling further support this conclusion [56],[57]. A recent report that Ful can induce HER ligands in ER+ breast cancer cells and thereby activate the PI3K/AKT pathway may explain the efficacy of the combination of Ful with the AKTi [58]. Our observation that the levels of AKT phosphorylation are increased in MCF7L xenograft tumors after two weeks of Ful treatment provides an additional rationale for combining AKT inhibition with Ful, a combination that should be tested in the clinic.

## Conclusions

This study shows that moderate PTEN reduction enhances multiple PI3K downstream signals, resulting in a global change of gene expression profile toward the luminal B subtype and endocrine resistance. Overcoming endocrine resistance by adding single or combined kinase inhibitors targeting mTOR, AKT, or MEK is a promising strategy but needs optimization based on PTEN levels and the type of endocrine agents used. Thus, the PTEN level may serve as a predictive marker for endocrine therapy and may also guide the design of combinatorial therapy. Among the single-agent kinase inhibitors, an AKT inhibitor best potentiates the efficacy of fulvestrant both *in vitro* and *in vivo*, providing a rationale for evaluating this combination in patients with ER+ tumors with reduced PTEN.

## Authors' contributions

XF, RS, and CKO conceived and designed the study, analyzed the data and wrote the manuscript. XF, VK, and SN performed most of the experiments. CJC, TW, and SGH performed biostatistics and bioinformatics work and helped to analyze the data. MS, MG, GM, AN, and MFR helped in performing mouse model experiments and data analysis. SH, AC, and CG conducted the pathology assessment. KLK and TFW provided the lentivirus inducible system and data interpretation. NCB and AJ performed the live animal imaging and data analysis. LMH, PA, PS, and JWG performed the RNA-seq and helped with data analysis. SMG, PDS, BRD, and TK participated in animal experimental design and data analysis, provided the drugs, and critically reviewed the manuscript. AVL and GBM substantially contributed to the design of the study and manuscript review. All authors read and approved the final manuscript.

## Additional files

## Electronic supplementary material

Additional file 1: Figure S1.: PTEN shRNA sequences were verified in breast cancer cells sorted by different eGFP intensity. **(A)** BT483 cells were infected with pGIPZ-shPTEN lentivirus and sorted by low (L)/high (H) intensity of eGFP. **(B)** Western blotting of PTEN in BT483 cells with four different shRNA sequences from pGIPZ vector (V2LHS series). The shRNA of luciferase (shLuc) was used as the negative control. **(C)** MCF7L cells were infected with pINDUCER-shPTEN lentivirus and sorted the same way as in A. **(D)** MCF7L-shPTEN_#1 cells with one of the verified shPTEN sequences were sorted for L/H- eGFP intensity and induced by Dox for two or five days. MCF7L-shLuc cells were used as knockdown (KD) control. Cell lysates were subjected to Western blotting of PTEN and β-actin. (TIFF 6 MB)

Additional file 2: Figure S2.: The effect of consistent KD by two PTEN shRNAs on MCF7L cells was not seen in the non-specific shRNA control. **(A)** PTEN mRNA levels were measured by qRT-PCR in MCF7L-shPTEN cells with two different shRNA sequences (#1 and #2). **(B),** MCF7L-shLuc cell lysates under -/+Dox were subjected to Western blotting as indicated. **(C)**, MCF7L-shLuc cells were cultured in phenol-red free (PRF) medium with 5% charcoal-stripped (CS)-FBS and -/+Dox for three days before being subjected to E2 (1 nM), ED, Tam (100 nM), or Ful (100 nM). Cell growth (%) was normalized to E2 controls (-/+Dox). Bonferroni *post hoc* comparison was performed within each treatment (-/+Dox) (N.S., not significant). (TIFF 1 MB)

Additional file 3: Figure S3.: Reduced PTEN causes decreased ER and its regulated genes, and is associated with the luminal B subtype of breast cancer. **(A)** The mRNA levels of ER and its regulated genes were measured by qRT-PCR in MCF7L-shPTEN cells in -/+Dox for three days. *GAPDH* mRNA levels were used as internal control. Gene expression in cells with the ED condition was used as a normalization control (set as 1). **(B)** Box plot shows the PTEN mRNA levels in the luminal A and B tumors from datasets of TCGA and Compendium. The mean value ± standard deviation of all samples in each subtype is marked on the box plot in red. All the pairwise comparisons were performed by Bonferroni *post hoc* test (^*^P <0.05, ^**^P <0.01, ^***^P <0.001). (TIFF 953 KB)

Additional file 4: Figure S4.: PTEN mRNA levels are not correlated with *PIK3CA* mutations in ER+ breast cancer. A total of 349 ER+ luminal tumors from the TCGA dataset were ranked from high to low PTEN mRNA levels (log2 transformed and median-centered). The status of *PIK3CA* gene mutations (red line indicates mutated) was aligned to the corresponding tumors. Spearman’s test of the correlation of PTEN mRNA levels and *PIK3CA* mutations was applied (N.S., not significant). (TIFF 483 KB)

Additional file 5: Figure S5.: PTEN KD decreases endocrine sensitivity in shPTEN cell models. **(A)** PTEN KD attenuated the blocking of S-phase entry by anti-estrogen treatment in MCF7L-shPTEN cells. Cell cycle distribution was measured in MCF7L-shPTEN cells under -/+Dox and endocrine treatment for three days. Cell population in G1 phase was compared between -/+Dox in each treatment group. **(B)** Colonies of MCF7L-shPTEN cells under -/+Dox and endocrine treatment for three weeks were stained by crystal violet. Quantification of colony formation was performed by ImageJ software. **(C)** Tumorspheres of BT483-shPTEN cells under -/+Dox and endocrine treatment for two weeks were scanned and quantified by cell cytometry (Celigo). Inset image shows the tRFP signal under fluorescence scanning. Scale bar, 100 μm. The Bonferroni *post hoc* test was used for all pairwise comparisons between -/+Dox (^*^P <0.05, ^**^P <0.01), or between E2 and anti-estrogen groups (^#^*P* <0.05). (TIFF 4 MB)

Additional file 6: Figure S6.: The optimized PTEN IHC protocol was verified in a cell pellet index array. **(A)** MCF7L-shPTEN cells were cultured in medium containing Dox (1 μg/ml) for different days, or a dose range of Dox for seven days, before being fixed in 10% neutral-buffered formalin and then embedded in paraffin. The processed cell pellets were organized in one slide (index array) as shown. **(B)** Representative IHC images for PTEN staining in the index array. Scale bar, 200 μm. (TIFF 3 MB)

Additional file 7: Figure S7.: Kinase inhibitors at the single dose used in cell growth assays effectively suppress the corresponding downstream signaling. MCF7L-shPTEN cells were grown in PRF medium with 5% CS-FBS for three days and then treated with DMSO (control), mTORi (0.2 μm), AKTi (1 μm), or MEKi (1 μm) for 3 hours or 24 hours. The cell lysates were harvested for the measurement of the phosphoproteins by Western blotting. (TIFF 689 KB)

Additional file 8: Figure S8.: Statistical analysis for drug interactions was performed by the Min test as described in Methods and the results are presented by heat maps showing the color-scaled *P* values for each drug combination matrix under ED **(A-C)** or Tam **(D-F)**. (TIFF 7 MB)

Below are the links to the authors’ original submitted files for images.

Authors’ original file for figure 1

Authors’ original file for figure 2

Authors’ original file for figure 3

Authors’ original file for figure 4

Authors’ original file for figure 5

Authors’ original file for figure 6

## Abbreviations

AKT: protein kinase B
CS-FBS: charcoal-stripped-FBS
Dox: doxycycline
E2: β-estradiol
ED: estrogen deprivation
eGFP: enhanced GFP
ER: estrogen receptor α
FBS: fetal bovine serum
Ful: fulvestrant
GFRs: growth factor receptors
HER2: human epidermal growth factor receptor 2
IHC: immunohistochemical
KD: knockdown
LTED: long-term estrogen deprivation
MAPK: mitogen-activated protein kinase
MEK: mitogen-activated protein kinase kinase
mTOR: mammalian target of rapamycin
PI3K: phosphatidylinositol 3-kinase
PRF: phenol-red free
PTEN: phosphatase and tensin homolog
RPPA: reverse-phase protein array
shLuc: luciferase shRNA
shRNA: short hairpin RNA
Tam: tamoxifen
TCGA: The Cancer Genome Atlas
tRFP: turbo-RFP
TTP: time to tumor progression
TTR: time to tumor regression
WT: wild-type
