# Supplementary figures and images for: Overcoming endocrine resistance due to reduced PTEN levels in estrogen receptor-positive breast cancer by co-targeting mammalian target of rapamycin, protein kinase B, or mitogen-activated protein kinase kinase
Source: Breast Cancer Res. 2014 Sep 11;16:430. doi: 10.1186/s13058-014-0430-x (PMC4303114; doi:10.1186/s13058-014-0430-x)

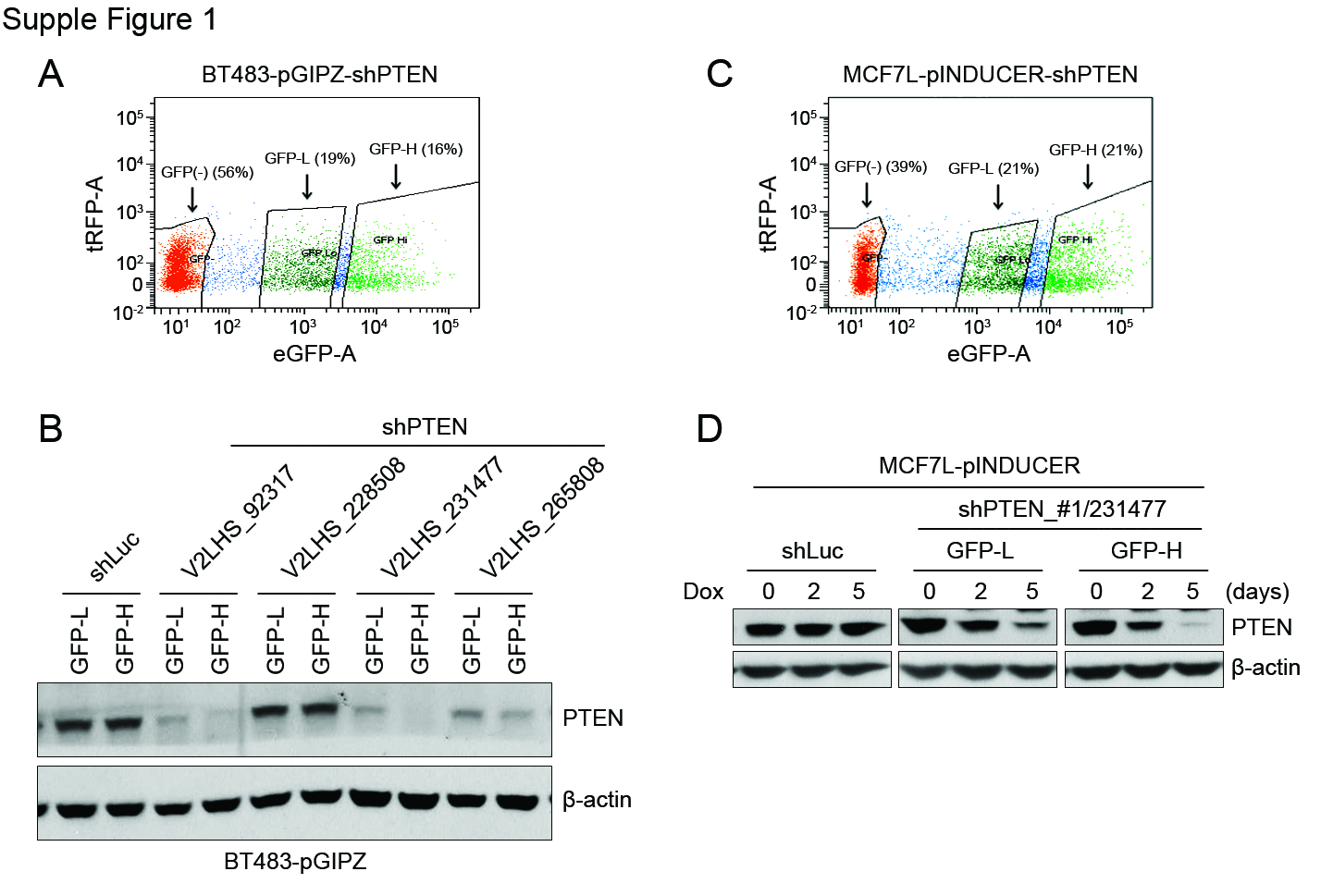

Supplement: Supplementary file 1 — Additional file 1: Figure S1.: PTEN shRNA sequences were verified in breast cancer cells sorted by different eGFP intensity. (A) BT483 cells were infected with pGIPZ-shPTEN lentivirus and sorted by low (L)/high (H) intensity of eGFP. (B) Western blotting of PTEN in BT483 cells with four different shRNA sequences from pGIPZ vector (V2LHS series). The shRNA of luciferase (shLuc) was used as the negative control. (C) MCF7L cells were infected with pINDUCER-shPTEN lentivirus and sorted the same way as in A. (D) MCF7L-shPTEN_#1 cells with one of the verified shPTEN sequences were sorted for L/H- eGFP intensity and induced by Dox for two or five days. MCF7L-shLuc cells were used as knockdown (KD) control. Cell lysates were subjected to Western blotting of PTEN and β-actin. (TIFF 6 MB) [file 13058_2014_430_MOESM1_ESM.tiff]

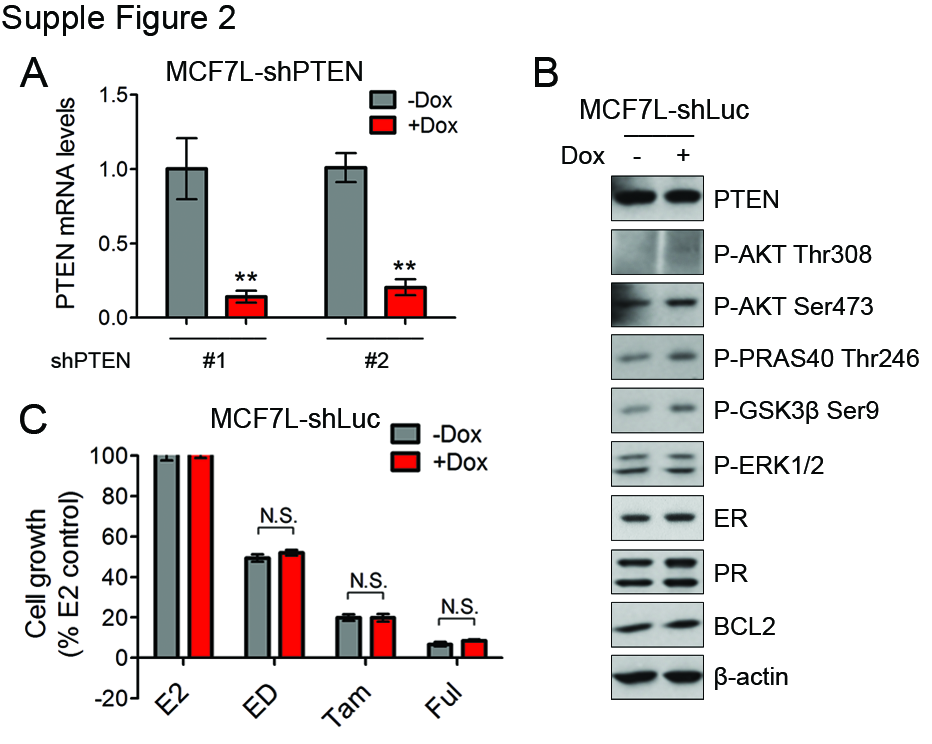

Supplement: Supplementary file 2 — Additional file 2: Figure S2.: The effect of consistent KD by two PTEN shRNAs on MCF7L cells was not seen in the non-specific shRNA control. (A) PTEN mRNA levels were measured by qRT-PCR in MCF7L-shPTEN cells with two different shRNA sequences (#1 and #2). (B), MCF7L-shLuc cell lysates under -/+Dox were subjected to Western blotting as indicated. (C), MCF7L-shLuc cells were cultured in phenol-red free (PRF) medium with 5% charcoal-stripped (CS)-FBS and -/+Dox for three days before being subjected to E2 (1 nM), ED, Tam (100 nM), or Ful (100 nM). Cell growth (%) was normalized to E2 controls (-/+Dox). Bonferroni post hoc comparison was performed within each treatment (-/+Dox) (N.S., not significant). (TIFF 1 MB) [file 13058_2014_430_MOESM2_ESM.tiff]

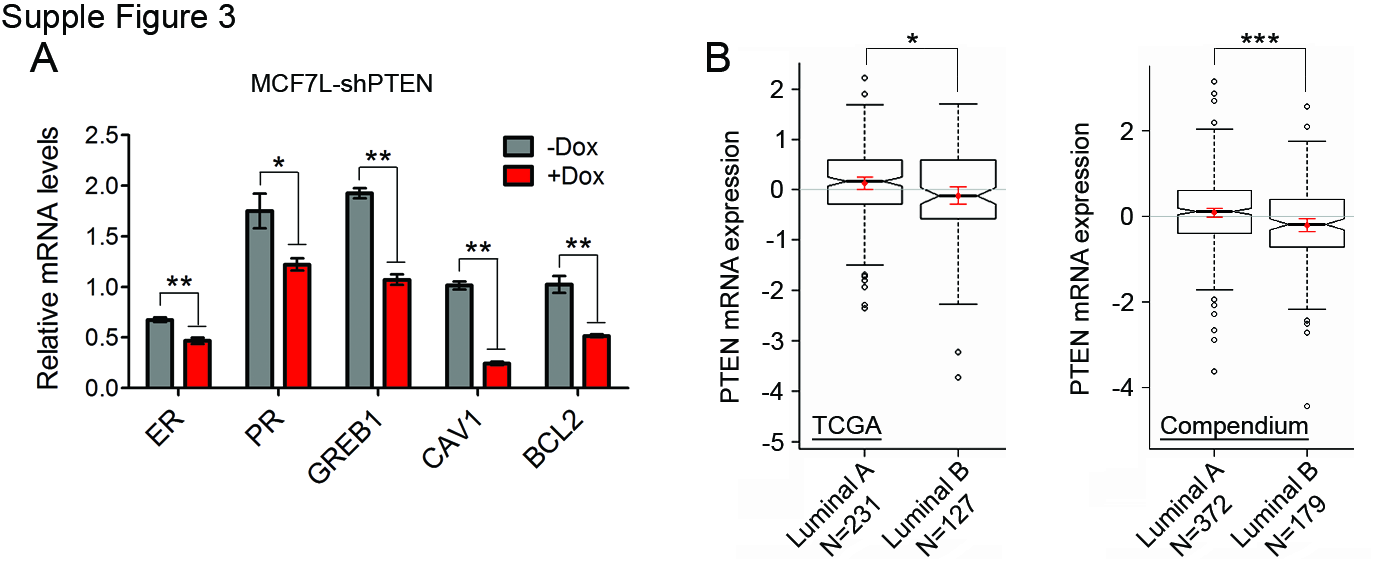

Supplement: Supplementary file 3 — Additional file 3: Figure S3.: Reduced PTEN causes decreased ER and its regulated genes, and is associated with the luminal B subtype of breast cancer. (A) The mRNA levels of ER and its regulated genes were measured by qRT-PCR in MCF7L-shPTEN cells in -/+Dox for three days. GAPDH mRNA levels were used as internal control. Gene expression in cells with the ED condition was used as a normalization control (set as 1). (B) Box plot shows the PTEN mRNA levels in the luminal A and B tumors from datasets of TCGA and Compendium. The mean value ± standard deviation of all samples in each subtype is marked on the box plot in red. All the pairwise comparisons were performed by Bonferroni post hoc test (*P <0.05, **P <0.01, ***P <0.001). (TIFF 953 KB) [file 13058_2014_430_MOESM3_ESM.tiff]

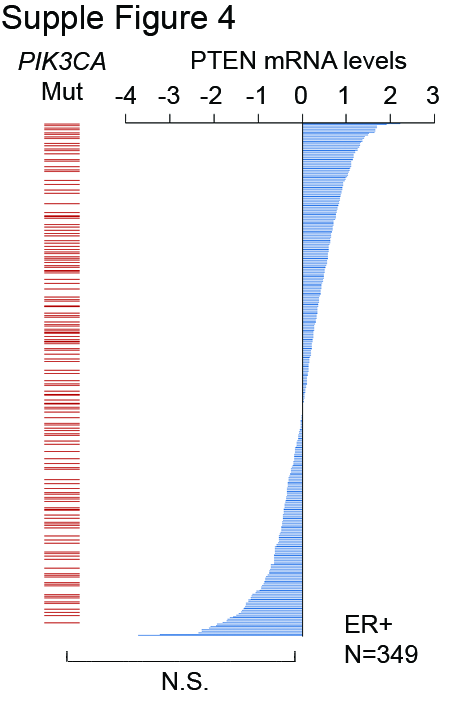

Supplement: Supplementary file 4 — Additional file 4: Figure S4.: PTEN mRNA levels are not correlated with PIK3CA mutations in ER+ breast cancer. A total of 349 ER+ luminal tumors from the TCGA dataset were ranked from high to low PTEN mRNA levels (log2 transformed and median-centered). The status of PIK3CA gene mutations (red line indicates mutated) was aligned to the corresponding tumors. Spearman’s test of the correlation of PTEN mRNA levels and PIK3CA mutations was applied (N.S., not significant). (TIFF 483 KB) [file 13058_2014_430_MOESM4_ESM.tiff]

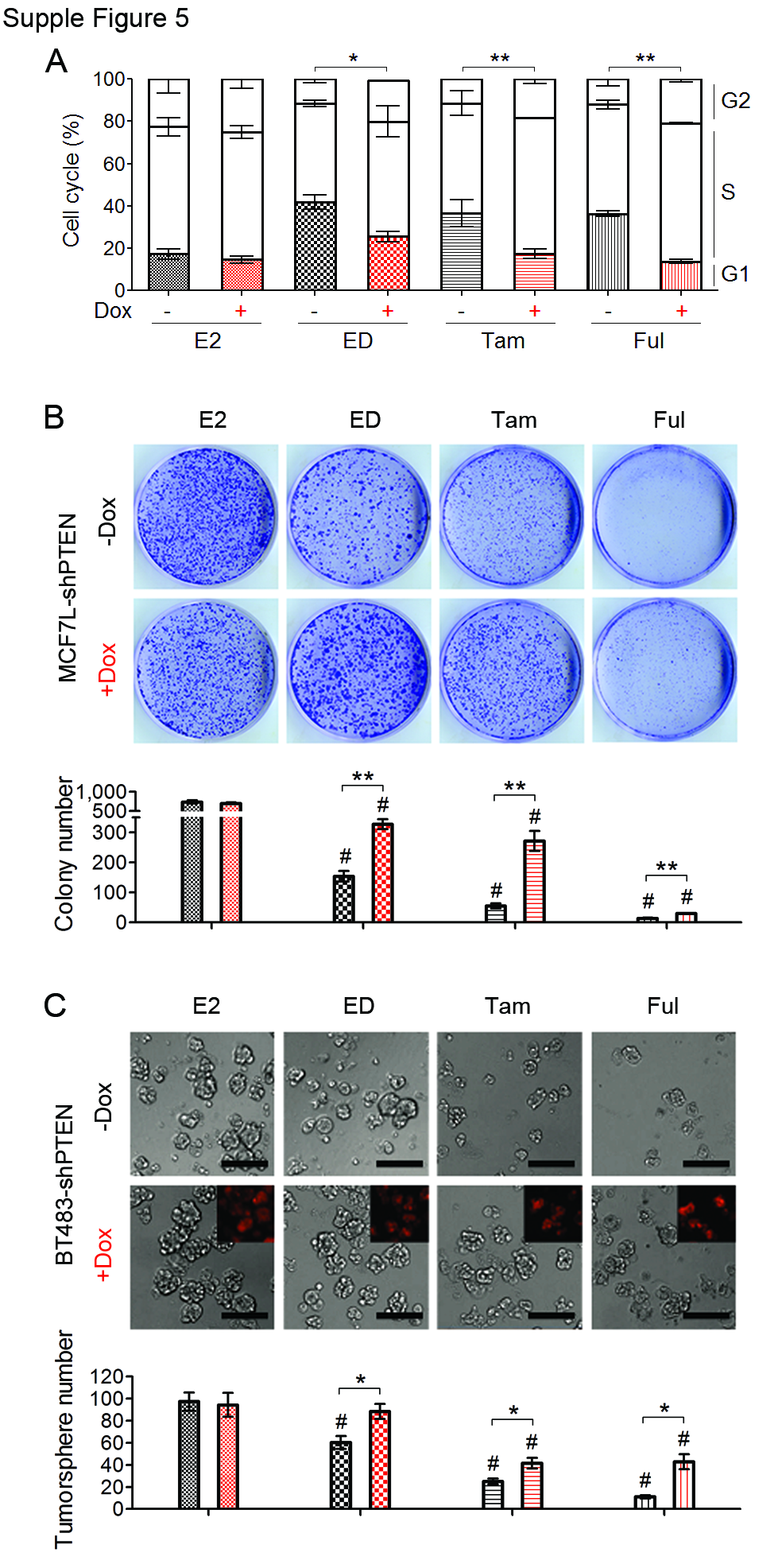

Supplement: Supplementary file 5 — Additional file 5: Figure S5.: PTEN KD decreases endocrine sensitivity in shPTEN cell models. (A) PTEN KD attenuated the blocking of S-phase entry by anti-estrogen treatment in MCF7L-shPTEN cells. Cell cycle distribution was measured in MCF7L-shPTEN cells under -/+Dox and endocrine treatment for three days. Cell population in G1 phase was compared between -/+Dox in each treatment group. (B) Colonies of MCF7L-shPTEN cells under -/+Dox and endocrine treatment for three weeks were stained by crystal violet. Quantification of colony formation was performed by ImageJ software. (C) Tumorspheres of BT483-shPTEN cells under -/+Dox and endocrine treatment for two weeks were scanned and quantified by cell cytometry (Celigo). Inset image shows the tRFP signal under fluorescence scanning. Scale bar, 100 μm. The Bonferroni post hoc test was used for all pairwise comparisons between -/+Dox (*P <0.05, **P <0.01), or between E2 and anti-estrogen groups (#P <0.05). (TIFF 4 MB) [file 13058_2014_430_MOESM5_ESM.tiff]

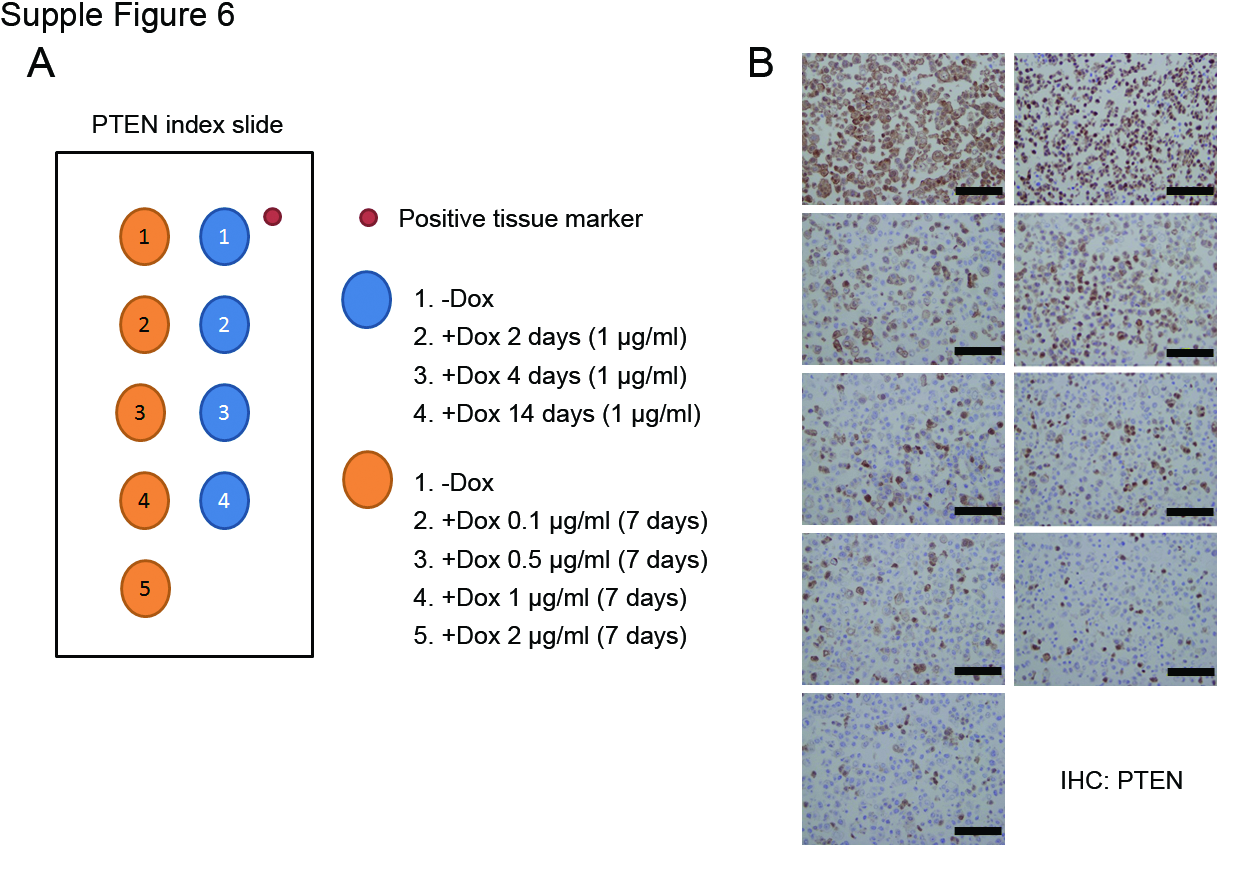

Supplement: Supplementary file 6 — Additional file 6: Figure S6.: The optimized PTEN IHC protocol was verified in a cell pellet index array. (A) MCF7L-shPTEN cells were cultured in medium containing Dox (1 μg/ml) for different days, or a dose range of Dox for seven days, before being fixed in 10% neutral-buffered formalin and then embedded in paraffin. The processed cell pellets were organized in one slide (index array) as shown. (B) Representative IHC images for PTEN staining in the index array. Scale bar, 200 μm. (TIFF 3 MB) [file 13058_2014_430_MOESM6_ESM.tiff]

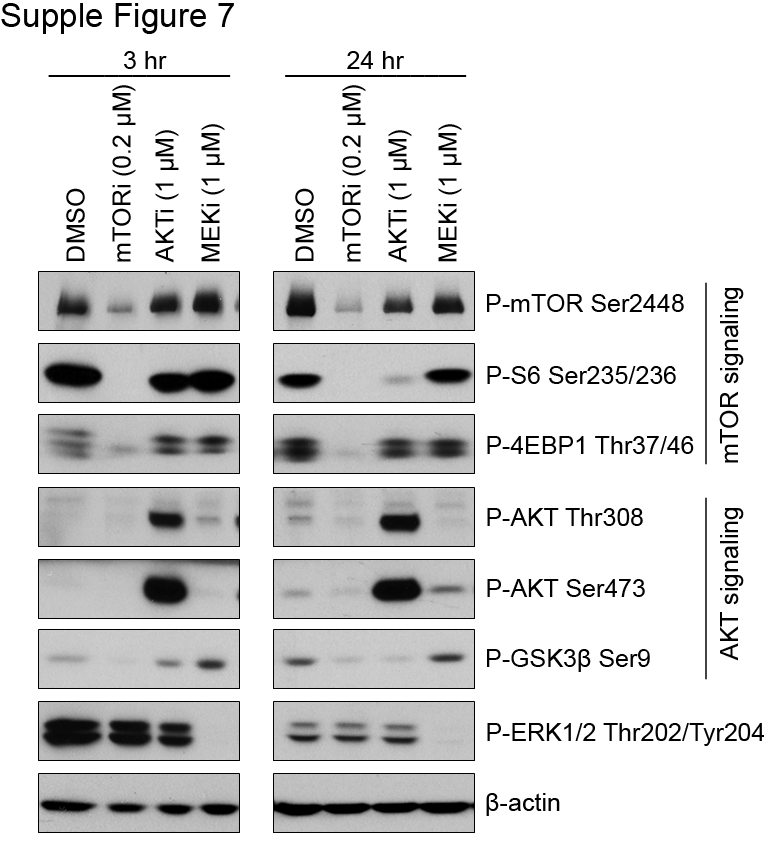

Supplement: Supplementary file 7 — Additional file 7: Figure S7.: Kinase inhibitors at the single dose used in cell growth assays effectively suppress the corresponding downstream signaling. MCF7L-shPTEN cells were grown in PRF medium with 5% CS-FBS for three days and then treated with DMSO (control), mTORi (0.2 μm), AKTi (1 μm), or MEKi (1 μm) for 3 hours or 24 hours. The cell lysates were harvested for the measurement of the phosphoproteins by Western blotting. (TIFF 689 KB) [file 13058_2014_430_MOESM7_ESM.tiff]

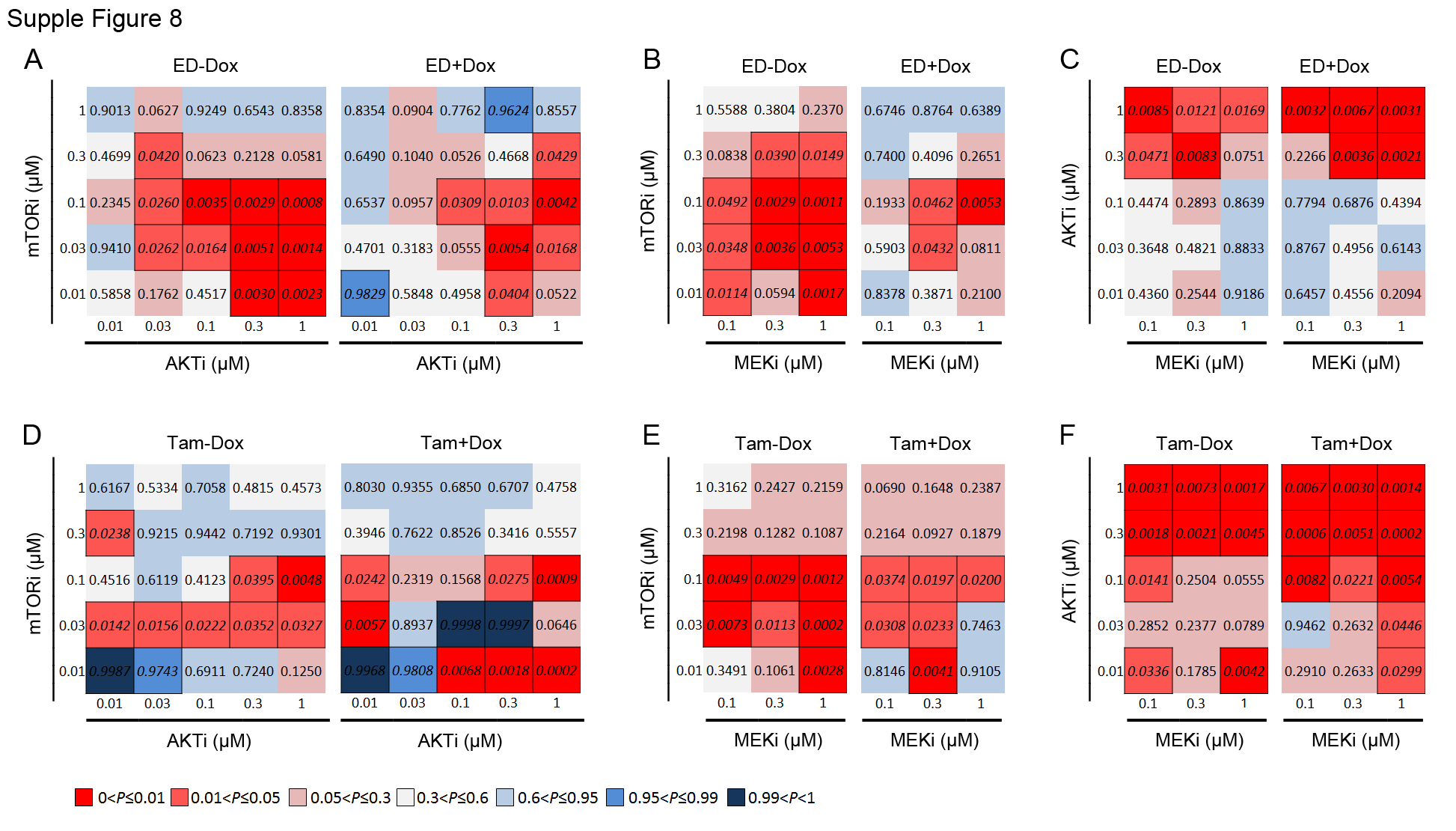

Supplement: Supplementary file 8 — Additional file 8: Figure S8.: Statistical analysis for drug interactions was performed by the Min test as described in Methods and the results are presented by heat maps showing the color-scaled P values for each drug combination matrix under ED (A-C) or Tam (D-F). (TIFF 7 MB) [file 13058_2014_430_MOESM8_ESM.tiff]

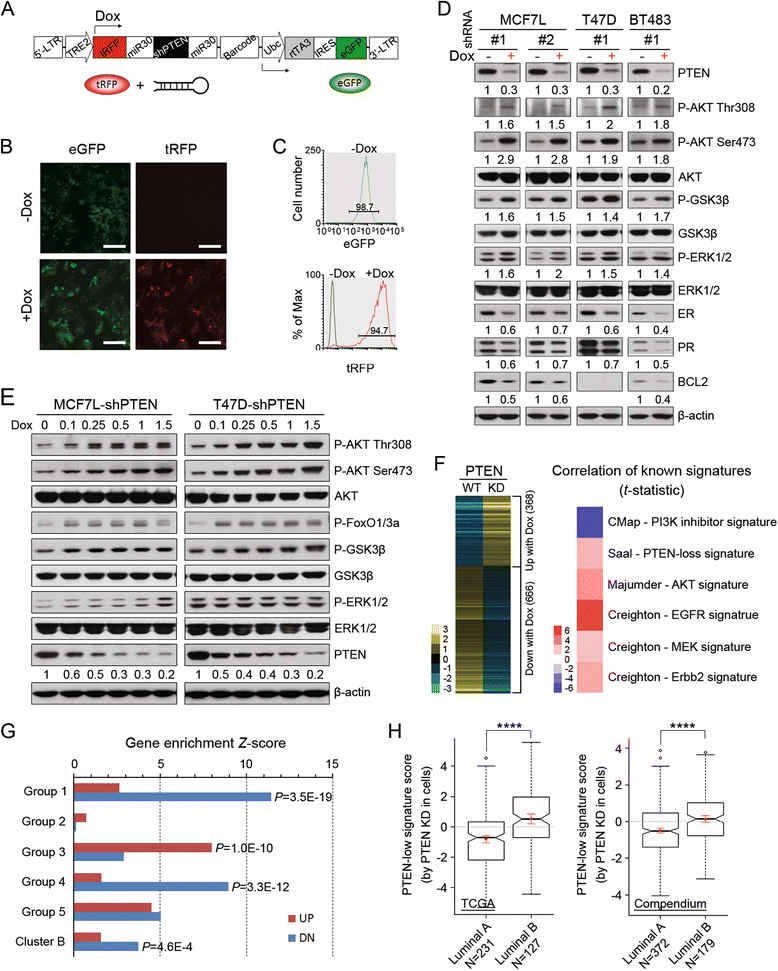

Supplement: Supplementary file 9 — Authors’ original file for figure 1 [file 13058_2014_430_MOESM9_ESM.gif]

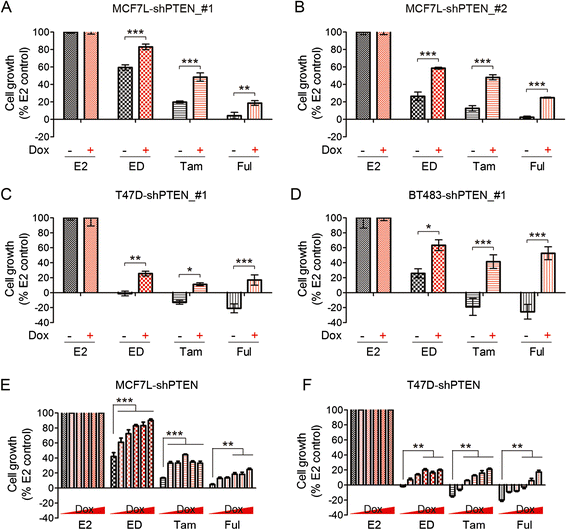

Supplement: Supplementary file 10 — Authors’ original file for figure 2 [file 13058_2014_430_MOESM10_ESM.gif]

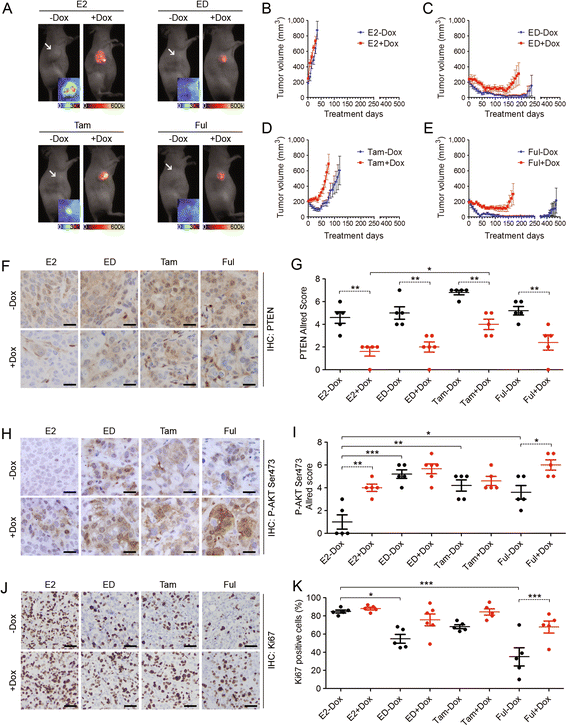

Supplement: Supplementary file 11 — Authors’ original file for figure 3 [file 13058_2014_430_MOESM11_ESM.gif]

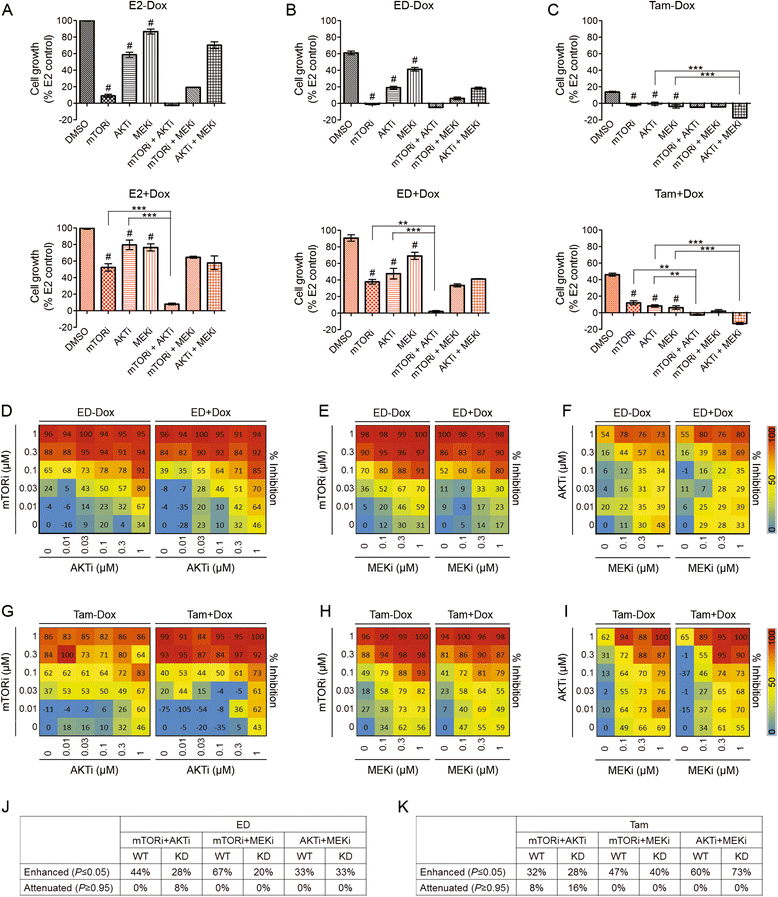

Supplement: Supplementary file 12 — Authors’ original file for figure 4 [file 13058_2014_430_MOESM12_ESM.gif]

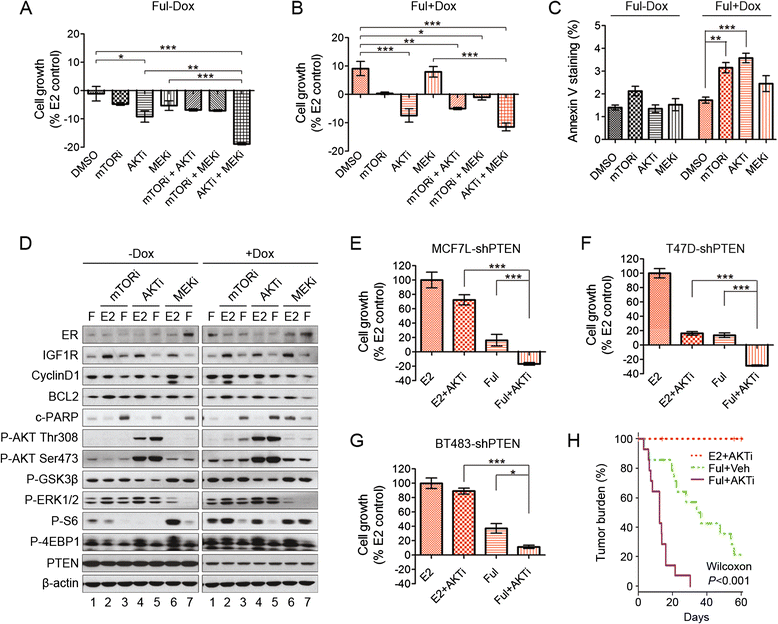

Supplement: Supplementary file 13 — Authors’ original file for figure 5 [file 13058_2014_430_MOESM13_ESM.gif]
